# Biaxial stretching of polytetrafluoroethylene in industrial scale to fabricate medical ePTFE membrane with node-fibril microstructure

**DOI:** 10.1093/rb/rbad056

**Published:** 2023-06-02

**Authors:** Gang Wang, Yusheng Feng, Caiyun Gao, Xu Zhang, Qunsong Wang, Jie Zhang, Hongjie Zhang, Yongqiang Wu, Xin Li, Lin Wang, Ye Fu, Xiaoye Yu, Deyuan Zhang, Jianxiong Liu, Jiandong Ding

**Affiliations:** State Key Laboratory of Molecular Engineering of Polymers, Department of Macromolecular Science, Fudan University, Shanghai 200438, China; R&D Center, Lifetech Scientific (Shenzhen) Co., Ltd., Shenzhen 518057, China; R&D Center, Lifevalve Medical Scientific Co., Ltd., Shenzhen 518057, China; R&D Center, Lifetech Scientific (Shenzhen) Co., Ltd., Shenzhen 518057, China; State Key Laboratory of Molecular Engineering of Polymers, Department of Macromolecular Science, Fudan University, Shanghai 200438, China; R&D Center, Lifetech Scientific (Shenzhen) Co., Ltd., Shenzhen 518057, China; R&D Center, Lifevalve Medical Scientific Co., Ltd., Shenzhen 518057, China; State Key Laboratory of Molecular Engineering of Polymers, Department of Macromolecular Science, Fudan University, Shanghai 200438, China; R&D Center, Lifetech Scientific (Shenzhen) Co., Ltd., Shenzhen 518057, China; R&D Center, Lifevalve Medical Scientific Co., Ltd., Shenzhen 518057, China; State Key Laboratory of Molecular Engineering of Polymers, Department of Macromolecular Science, Fudan University, Shanghai 200438, China; R&D Center, Lifetech Scientific (Shenzhen) Co., Ltd., Shenzhen 518057, China; R&D Center, Lifevalve Medical Scientific Co., Ltd., Shenzhen 518057, China; State Key Laboratory of Molecular Engineering of Polymers, Department of Macromolecular Science, Fudan University, Shanghai 200438, China; R&D Center, Lifetech Scientific (Shenzhen) Co., Ltd., Shenzhen 518057, China; R&D Center, Lifevalve Medical Scientific Co., Ltd., Shenzhen 518057, China; State Key Laboratory of Molecular Engineering of Polymers, Department of Macromolecular Science, Fudan University, Shanghai 200438, China; State Key Laboratory of Molecular Engineering of Polymers, Department of Macromolecular Science, Fudan University, Shanghai 200438, China; R&D Center, Lifetech Scientific (Shenzhen) Co., Ltd., Shenzhen 518057, China; R&D Center, Lifetech Scientific (Shenzhen) Co., Ltd., Shenzhen 518057, China; State Key Laboratory of Molecular Engineering of Polymers, Department of Macromolecular Science, Fudan University, Shanghai 200438, China

**Keywords:** biomedical polymer, medical-grade raw material, expanded polytetrafluoroethylene, olive roller, polymer processing, biaxial stretching

## Abstract

Expanded polytetrafluoroethylene (ePTFE) is promising in biomedical fields such as covered stents and plastic surgery owing to its excellent biocompatibility and mechanical properties. However, ePTFE material prepared by the traditional biaxial stretching process is with thicker middle and thinner sides due to the bowing effect, which poses a major problem in industrial-scale fabrication. To solve this problem, we design an olive-shaped winding roller to provide the middle part of the ePTFE tape with a greater longitudinal stretching amplitude than the two sides, so as to make up for the excessive longitudinal retraction tendency of the middle part when it is transversely stretched. The as-fabricated ePTFE membrane has, as designed, uniform thickness and node-fibril microstructure. In addition, we examine the effects of mass ratio of lubricant to PTFE powder, biaxial stretching ratio and sintering temperature on the performance of the resultant ePTFE membranes. Particularly, the relation between the internal microstructure of the ePTFE membrane and its mechanical properties is revealed. Besides stable mechanical properties, the sintered ePTFE membrane exhibits satisfactory biological properties. We make a series of biological assessments including *in vitro* hemolysis, coagulation, bacterial reverse mutation and *in vivo* thrombosis, intracutaneous reactivity test, pyrogen test and subchronic systemic toxicity test; all of the results meet the relevant international standards. The muscle implantation of the sintered ePTFE membrane into rabbits indicates acceptable inflammatory reactions of our sintered ePTFE membrane fabricated on industrial scale. Such a medical-grade raw material with the unique physical form and condensed-state microstructure is expected to afford an inert biomaterial potentially for stent-graft membrane.

## Graphical abstract

**Figure:**
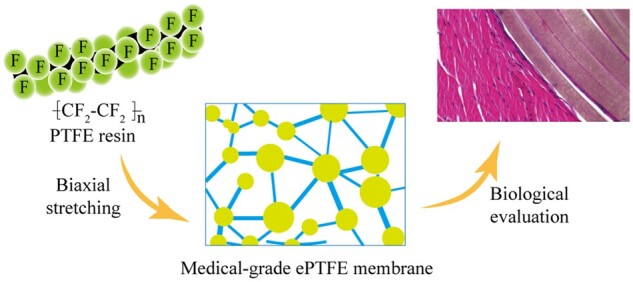

## Introduction

Polytetrafluoroethylene (PTFE) is a homopolymer of tetrafluoroethylene with a highly regular molecular chain—C–C bonds as the main chain, stable C–F bonds distributed on both sides, and fluorine atoms completely symmetrical in the main chain with crystallinity up to 99%. Since synthesized in 1938, it has been much developed and widely used in various fields such as chemical industry, machinery, aviation etc. [1–4]. PTFE has good mechanical properties, low friction coefficient, cold and heat resistance, and aging resistance. In addition, the close shielding of the C–F bond on the main chain protects the PTFE molecular chain from destruction. Therefore, PTFE hardly suffers from any material corrosion except reacting with molten alkali metals. While a variety of polymers have been applied in the biomedical field [5–8], PTFE distinguishes itself as its little immunity response and high stability [9–13].

In particular, a unique physical form of PTFE is expanded PTFE (ePTFE), which was invented in 1969 by Gore [14]. ePTFE is produced by processing starting from PTFE resin, leading to a network structure of connected microfibers. ePTFE inherits the excellent properties of PTFE, and forms fibers and pores during the molding process. The resultant node-fibril microstructure makes ePTFE tougher, and its breaking strength and elongation have been significantly improved compared with the corresponding PTFE raw material. This microstructure also makes ePTFE to attach to human tissues easily, and thus affords an ideal biological tissue substitute and one of the human implant materials [15–18]. Medical devices made of ePTFE have been used clinically in many aspects, such as oral surgery [19], nose correction [20, 21], kidney reconstruction [22] and covered vascular stent [23–25]. For example, Nakajima *et al*. [24] applied an ePTFE-covered stent to repair the deep femoral artery injury caused by femoral trochanteric fracture surgery. Piazza *et al.* [26] have compared the efficacy of ePTFE-covered stents and bare metal stents in the treatment of severe iliac artery obstructive lesions, and found that in some subgroups of lesions, covered stents provide a higher patency rate and can be used as the primary treatment. Chatterjee *et al*. [27] have treated radial artery perforation with ePTFE-covered coronary stents and found that covered stents may be an effective alternative to vascular surgery for perforation that does not respond to conservative treatment. In general, the covered stent is effective in opening stenosis and maintaining lumen patency, and can significantly improve the signs, symptoms and quality of life of patients [28, 29], among which, the ePTFE membrane also plays a key role in hemostasis, avoiding aneurysm and aortic dissection treatment.

So far, a few ePTFE-covered stents have been commercialized, such as Ankura, Viabahn and Aegis, which is beneficial for doctors and patients to make a choice. Nevertheless, the development of ePTFE-covered stents still remains challenging [14, 30, 31]. One of the problems comes from an uneven ePTFE membrane resulting from the traditional processing, while uniform and good mechanical properties are the prerequisite for the application of biomaterials [32–34]. In most countries such as China, any medical-grade ePTFE membrane has never been produced. Therefore, the development of the processing of high-standard and high-quality ePTFE membranes in industrial scale is meaningful for us and the biomaterial field.

**Figure 1.**
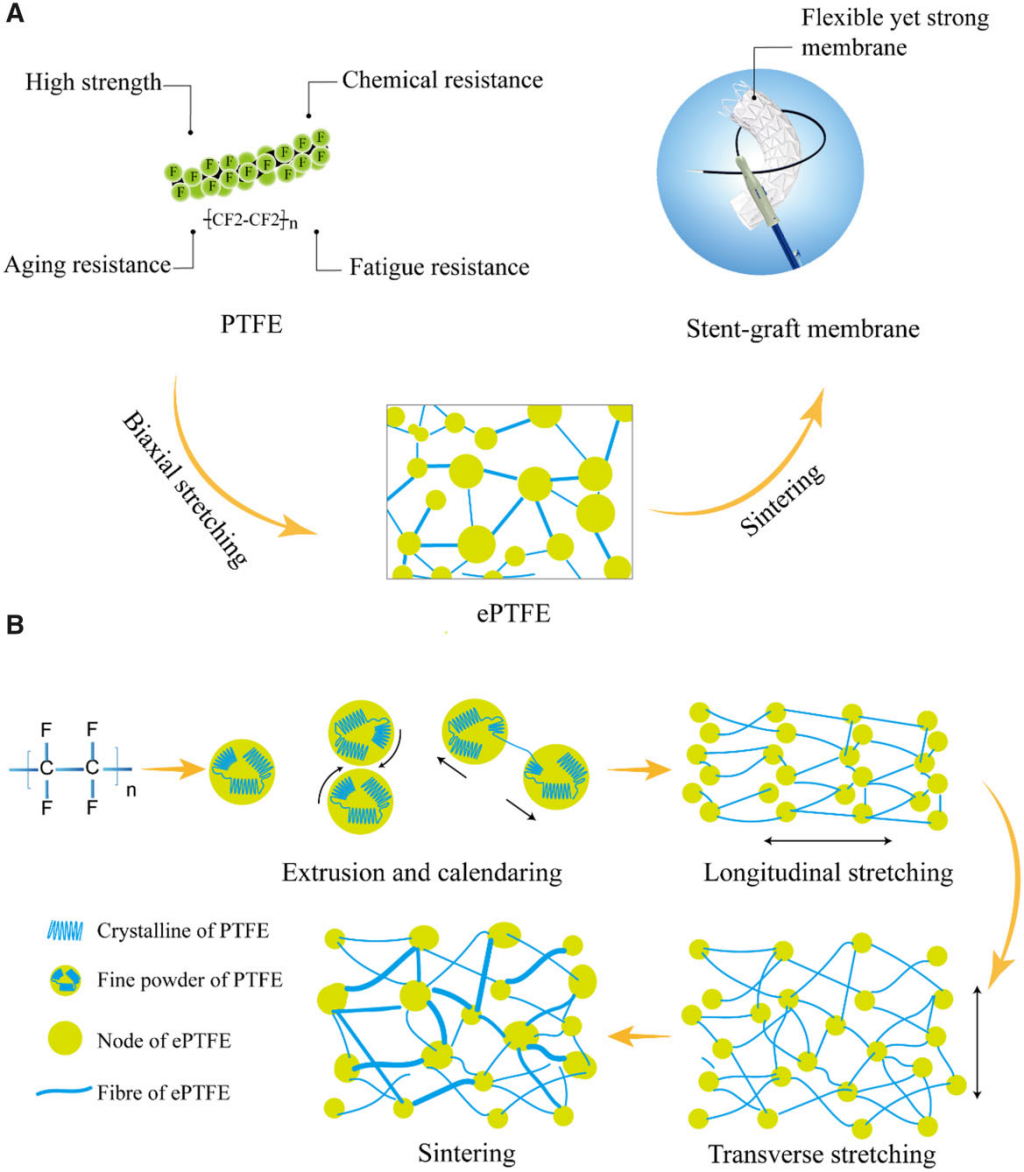
Schematic presentation of PTFE and ePTFE used for a medical device. (**A**) Illustration of the PTFE properties and the translation of PTFE to ePTFE by a biaxial stretching method as demonstrated in a stent-graft device. (**B**) Illustration of the principle to fabricate an ePTFE membrane. Fibrils are formed by the friction between the sheet-like crystals in the fine powder particles during the process of extrusion and calendaring, and the formation of a ‘node-fibril’ structure during the process of longitudinal stretching and transverse stretching.

The procedures of an ePTFE membrane generally contain mixing PTFE resin powder and an extrusion agent, extrusion molding, calendaring, biaxial stretching, curing and sintering (heat setting), and cooling [35–39]. We present the key aspects of ePTFE schematically in Fig. 1.

In the fabrication of ePTFE from PTFE resin, a membrane is first prepared after extruding and calendaring. Then, a longitudinal retraction force is exposed on the membrane during the longitudinal stretching. In the subsequent transverse stretching, both sides of the ePTFE membrane are fixed, resulting in a more serious longitudinal retraction in the middle area of the membrane with the increase of the transverse stretching ratio. Therefore, in the traditional biaxial stretching process, the tensile degree along the longitudinal middle area is less than that at both sides, resulting in uneven membrane thickness and different mechanical properties of each part.

Herein, we improve the biaxial stretching by designing an olive-shaped winding roller during the longitudinal stretching, which will be described in detail in ‘Results’ section. This new winding roller can make the middle of the ePTFE tape have a larger stretching amplitude relative to both sides during the longitudinal stretching, to compensate for the larger longitudinal retraction tendency of the ePTFE tape along the middle part in the subsequent transverse stretching. By this way, we have achieved relatively uniform membrane thickness. In addition, we have redesigned a clean workshop to assist in production to ensure the medical-grade requirements.

As an ePTFE membrane for a medical application, its physical properties have been accessed, and a series of biological characteristics of sintered ePTFE membranes have been performed. Both hemolysis and cytotoxicity results meet the requirements of International Organization for Standardization (ISO). We have also carried out thrombosis test, bacterial reverse mutation test, intracutaneous reactivity test, pyrogen test and subchronic systemic toxicity test to access its biocompatibility following the relevant medical device standards. In addition, we have implanted the ePTFE membrane into rabbits to examine the inflammatory responses of such a medical-grade product fabricated in industrial scale.

## Materials and methods

### Fabrication of ePTFE membrane

The PTFE resin powder (DAKIN, Japan) and lubricant (isomeric alkane solvent oil, Exxon Mobile, USA) were mixed, resulting in a homogeneous paste. The mixture was then filtered to remove large particles, forming a macroscopically homogeneous PTFE paste. The pasty PTFE material was pressed into a billet, then the billet was extruded into a rod-like bar. The extruded rod-like bar was calendared into a PTFE tape.

The prepared PTFE tape was stretched longitudinally in an oven, rolled up with a common roller or an olive roller. The first-stretched sample was stretched transversely in a tunnel oven to obtain the prefabricated product.

The prefabricated product was sintered (heat-set) in the tunnel oven and cooled in air after leaving the oven. Finally, the product was rolled up to obtain a biaxially stretched ePTFE membrane.

### Physical properties of ePTFE membranes

The tensile test of the ePTFE membrane was carried out in universal testing machine (LD22.503, LSI, Shanghai). Nine specimens were sampled in the transverse direction, and five along the longitudinal direction, as schematically presented in Supplementary Fig. S1. The tensile properties were measured with the test machine at the speed of 50 mm/min until the thin membrane broke. Use an automatic thickness gauge to measure its thickness.

The morphology of the membrane was observed with a field-emission scanning electron microscope (JSM-6510, JEOL, Japan) under 2 kV accelerating voltage. Prior to observation, each specimen was sputtered with gold at 10 mA for 90 s.

The thermal properties of ePTFE membranes were measured with a differential scanning calorimeter (Mettler, Switzerland). The temperature range was set between 40 and 400°C, and the heating rate was set as 10°C/min following the protocol in the literature [40].

### Fabrication of sintered membranes

The biaxially stretched ePTFE membrane was covered on a SUS304 mold bar with a diameter of 20 mm for seven to eight layers equal to the quantity of layers of the stent-graft as described in our previous work [41]. And then this assembly was placed in an oven and undergone a heat treatment, which is similar to the stent-graft fabrication. The layers of ePTFE were bonded to each other to form a cylindrical sintered ePTFE membrane.

### Cell viability

We employed an assay of 3-(4,5-dimethylthiazol-2-yl)-2,5-diphenyltetrazolium bromide (MTT) to access cell viability. Sodium dodecyl sulfate (SDS), high-density polyethylene (HDPE) and medium were used as positive, negative and blank control groups of cytotoxicity, respectively. Mouse fibroblasts (L929 cells) were cultured in the cell culture medium with SDS or the extracts at 37°C for 72 h. For each group, *n *=* *5. The absorbance of optical density at 570 and 630 nm wavelengths were detected to reflect the relative cell viability. The relative growth rate is defined by the absorbance of the test group over that of the blank control.

### Hemolysis

We added normal saline (0.9% sodium chloride) to the test tube with sintered ePTFE membrane samples as the test group, normal saline as the negative control group, distilled water as the positive control group. For each group, *n *=* *3. Diluted anticoagulated rabbit blood was added to each group, and centrifuged after incubation. The supernatant was detected at a wavelength of 545 nm. The absorbance was used to calculate the hemolytic rate.

### Coagulation

Sintered ePTFE membrane samples and platelet-poor plasma (PPP) were added into a polypropylene tube as the experimental group. The positive control group was added with glass beads and PPP, the negative reference sample group was added with HDPE and PPP, and the blank control group was added with PPP. For each group, *n *=* *3. The clotting time was recorded in a hemagglutination analyzer test.

### Thrombosis

The thrombogenic properties of sintered ePTFE membranes were evaluated by orthotopic implantation of the jugular vein in a canine model. Experimental animals were anesthetized with an intravenous injection of sodium pentobarbital. The skin of the neck was incised and the jugular vein was isolated. Sintered ePTFE membranes with a length of ∼12 cm were implanted along the vein toward the heart. Sutures were used to close the socket while the extracorporeal portions of the sample were sutured to the animal skin tissue. An unobstructed intravascular flow should be ensured. It was left in the animal for 4 h.

Heparin was injected at 50 IU/kg 4 h after implantation. Animals were euthanized 5–15 min after systemic heparinization. The samples were removed after dissection, and the samples and controls were washed in normal saline and fixed in 2.5% glutaraldehyde. Thrombus formation was observed under scanning electron microscopy (SEM) (JSM 6510, Nippon Electronic Co., Ltd, Japan).

### Bacterial reverse mutation test

Under aseptic operating conditions, a 66 cm^2^ sintered ePTFE membrane was placed in a sterile glass bottle and immersed at a 6 cm^2^/ml extraction ratio with the addition of 11 ml dimethylsulfoxide (DMSO) for 72 h at 50°C. DMSO was used as a negative control, and mutagen was used as a positive control.

The extracts were divided into activated and nonactivated groups and mixed with 0.1 ml of bacterial and 2 ml of broth medium, respectively. S9 solution (rat liver S9, Moltox, USA) was added to the activated group, and phosphate buffer saline was added to the nonactivated group. Negative and positive controls were plated identically. The results were observed after all plates were incubated at 37°C for 48–72 h, and the number of revertant mutant colonies in each dish was recorded.

### Intracutaneous reactivity test

After putting the samples into sterile glass bottles under sterile conditions, the extract was made at 50°C for 72 h according to the extraction ratio of 6 cm^2^/ml to prepare polar and non-polar extracts. After partial hair removal, the left and right sides of each rabbit spine were equally divided into five points on the upper left side for injection of polar extract, five points on the lower left side for injection of non-polar extract, five points on the upper right side for injection of polar control solution and five points on the lower right side for injection of non-polar control solution.

The skin reaction at each injection site was observed and recorded immediately, 24, 48 and 72 h after injection. The erythema and swelling at the injection site at each time point were observed and recorded.

### Pyrogen test

The extract was made at 50°C for 72 h, after putting the sample into a sterile glass bottle under sterile conditions and adding normal saline according to the extraction ratio of 6 cm^2^/ml. The extraction medium without test samples was prepared in the same way as the solvent control solution.

Three qualified experimental rabbits were screened the day before the test. The normal body temperature of the rabbits was measured before the test.

The extract preheated to 38°C was slowly injected from the rabbit ear vein at a dose of 10 ml/kg (body weight).

The body temperature was measured every 30 min for six consecutive measurements. The rising temperature of the rabbit’s body temperature was calculated by subtracting the normal body temperature from the highest of the six body temperatures.

### Subchronic systemic toxicity

The sintered ePTFE membrane was extracted for 72 h under the condition of 50 ± 2°C normal saline at the extraction ratio of 6 cm^2^/ml. The extract and normal saline were the experimental group and the control group, respectively. There were 10 rats of each sex in the experimental group and the control group. The experimental animals were weighed, marked and randomly divided. The difference in body weight of each sex animal shall not exceed ±20% of the average body weight.

In the experimental group, 10 male and 10 female rats experienced tail vein injections of the test solution for 28 consecutive days. The control group was injected with the control fluid in the same way. Animals were weighed weekly. All animal blood was collected via abdominal aorta for clinical pathological detection. Finally, the animals were euthanized and dissected for gross pathological observation. Collected tissue samples were fixed to make pathological sections for histopathological observation.

### Animal implantation studies of ePTFE membranes

The as-fabricated membrane was implanted into rabbits. After anesthesia, the dorsal skin was incised. Three samples of the sintered ePTFE membranes were implanted in the muscle along one side of the spine, while three control samples were implanted on the other side of the spine in each animal in the same way. Totally 20 animals were studied. After 1, 4, 12 and 26 weeks of implantation, animals were euthanized and implantation site muscles were sampled and processed for hematoxylin–eosin (HE) staining. The stained tissue pieces were observed under an optical microscope, and the tissue reaction extents around the implantation site were accessed, and compared between the test and control samples.

**Ethical declaration:** All the animal experiment protocols in this paper have been approved by the Institutional Animal Care and Use Committee (IACUC) of Shenzhen Advanced Animal Study Service Center, and the approval number is 180717A-PI22.

## Results

### Establishment of facilities to biaxially stretch PTFE in industrial scale

**Figure 2.**
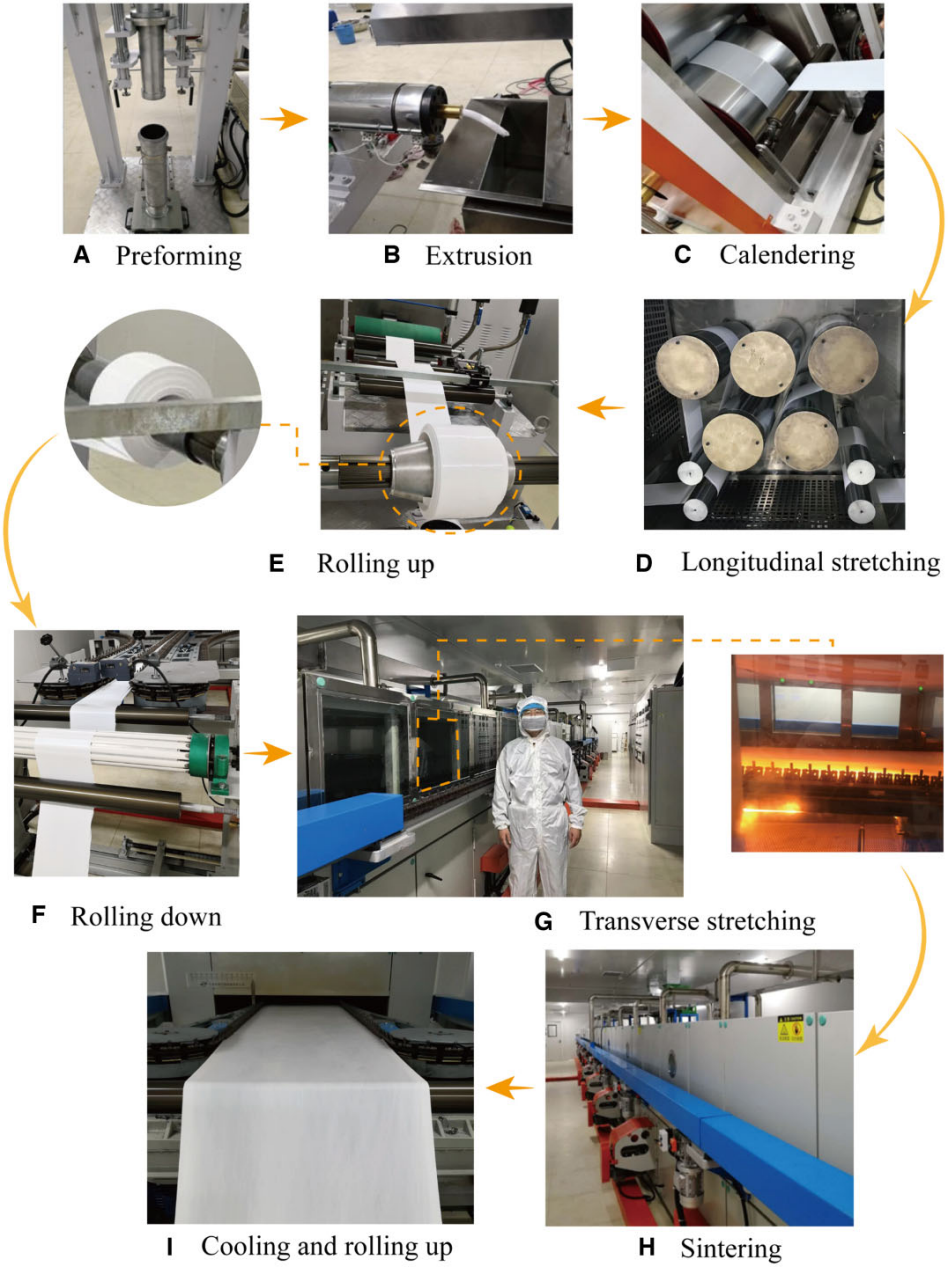
The manufacturing process of ePTFE membrane. (**A**) Preforming to compress the compound of PTFE fine powder and lubricant and remove the air in the compound. (**B**) Paste extrusion to make a rod by the press extruder machine from the preform. (**C**) Calendaring to make a primary tape from the rod by a twin-roller calendaring machine. (**D**) Longitudinal stretching. In this procedure, the primary tape was stretched several times by the rollers with different rotation speeds. (**E**) Rolling up the tape by a mandrel. The right is an olive roller and the left is a common cylinder roller. (**F**) Rolling down of the tape at the entry of the transverse stretching machine. (**G**) Transverse stretching. The left is an overview of the machine with the corresponding author of this article reflecting the size of our industry-scale facility, and the right is an inner view within the transverse stretching oven. (**H**) Sintering to fix the shape of the biaxial stretching of membrane. (**I**) Cooling and rolling up of the biaxial stretching ePTFE membrane.

The ePTFE membrane produced in this work was prepared in self-built production equipment, as shown in Fig. 2. The entire production process was carried out in a standard clean workshop. The preforming (Fig. 2A) was done with a hydraulic system with components such as solenoid change-over valve, pressure relief valve and speed control valve. The PTFE blank was ejected with the lower cylinder. An extrusion machine was used to further pressurize the PTFE blank (Fig. 2B). The working temperature and speed could be adjusted to extrude the PTFE rod with high density for calendaring, during which two motors were used to drive the rollers individually (Fig. 2C). The surface of each roller can be heated and controlled to form a primary PTFE tape between the rollers.

The longitudinal stretching device was placed in an oven and consisted of five heating towing rollers and four guiding rollers (Fig. 2D). The temperature and speed of each roller could be regulated independently. An observation hole was installed on the oven door. In the winding device (Fig. 2E), the winding tension was adjusted by a tension controller. In this work, an olive-shaped winding roller was selected.

Figure 2G and H shows transverse stretching and sintering devices. During the transverse stretching, the left and right ends of the ePTFE membrane were clamped. The device advanced gradually utilizing the precise chain drive. The transverse stretching and sintering processes in the tunnel oven were divided into preheating section, transverse stretching section, buffer section, sintering section and cooling section. The length and temperature of each section were adjustable. The biaxially stretched ePTFE membrane was rolled up after final cooling (Fig. 2I). The exhaust gas and oil fumes produced by the equipment were equipped with a device for collecting and filtering.

### Effects of transverse stretching using olive and common rollers on ePTFE membrane properties

**Figure 3.**
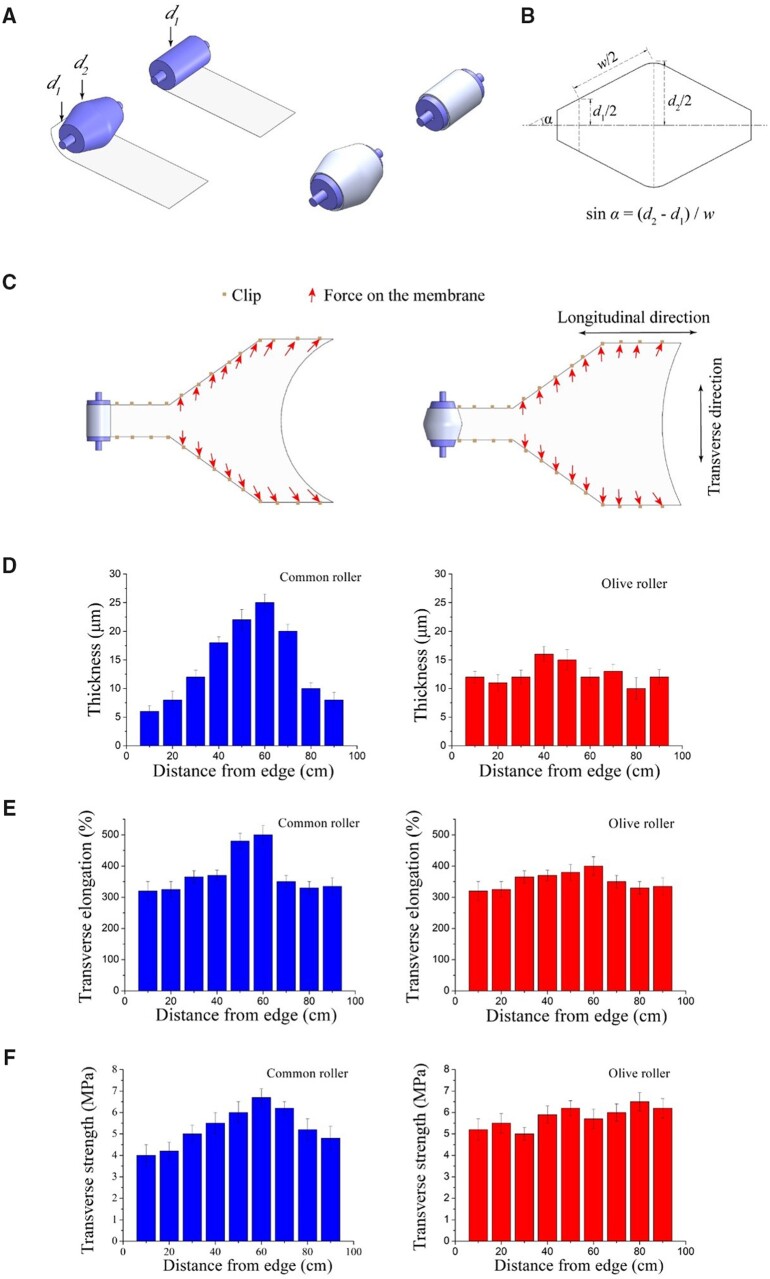
Diagram of using an olive roller to roll up the PTFE tape in the longitudinal stretching and then roll down in the transverse stretching to reduce the ‘bowing effect’ with the longitudinal contraction of the ePTFE membrane when both sides are grasped by the clips. (**A**) Comparative diagram of a common roller with a cylinder shape and an improved roller with an olive shape. The olive roller rolls up the PTFE tape on its surface in the rolling process, which will make the middle tape length longer than the side since the middle diameter (*d*_2_) is greater than the side diameter (*d*_1_). (**B**) An axial section diagram of an olive roller. Where ‘*α*’ is the angle between the roller surface and the axis and ‘*w*’ is the width of the tape wound around the roller. The ratio of the middle length to the edge length of the tape wound on the olive roller is *d*_2/_*d*_1_. (**C**) Comparative diagram of an olive roller and a common roller during rolling down in the transverse stretching process. During the transverse stretching, because the edges of the membrane are fixed by the clips and the longitudinal shrinkage of the membrane, the middle of the membrane will retract and form an arch shape at the beginning of the membrane (bowing effect). using an olive roller can reduce the bowing effect in transverse stretching because it can relieve the longitudinal contraction in the middle of the membrane. (**D**) The effect on the membrane thickness when stretching with a common roller and an olive roller. The transverse width of the membrane is 100 cm. A total of nine sampling points are along one side to the other side in the transverse direction. (**E**) The effect on the membrane transverse elongation when stretching with a common roller and an olive roller. (**F**) The effect on the membrane transverse strength when stretching with a common roller and an olive roller.

The principle of our introduction of an olive roller is schematically presented in Fig. 3A–C. The physical properties of the resultant ePTFE membranes were measured after transverse stretching on an olive roller and a common roller. According to Fig. 3D, when a common roller was used for transverse stretching, the resultant ePTFE membrane thickness was not uniform—the middle was thick while both sides were thin. The thickness range was 15.5 ± 9.5 μm with a variation of up to 19 μm. In contrast, the introduction of an olive roller into the transverse stretching led to a considerable improvement in thickness uniformity, with the overall fluctuation remaining within 10 μm.

We carried out a series of tensile tests of the as-fabricated ePTFE membranes with data shown in Fig. 3E and F. Common rollers resulted in site-dependent transverse elongation and strength, consistent with the variation of thickness results and poor uniformity. In contrast, when the olive roller was used for transverse stretching, the transverse elongation of the ePTFE membrane was maintained at 360 ± 40% and the transverse tensile strength was 5.75 ± 0.75 MPa. In general, with no performance loss in terms of thickness, the transverse elongation and transverse tensile strength, the ePTFE membrane stretched transversely with olive rollers exhibited significantly higher uniformity than the membrane prepared with common rollers. Good uniformity of the membrane can provide a safety guarantee during its use, and decrease the possibility of structural damage and longer service life.

### Effect of mass ratio of lubricant to PTFE powder on the performance of ePTFE membranes

**Figure 4.**
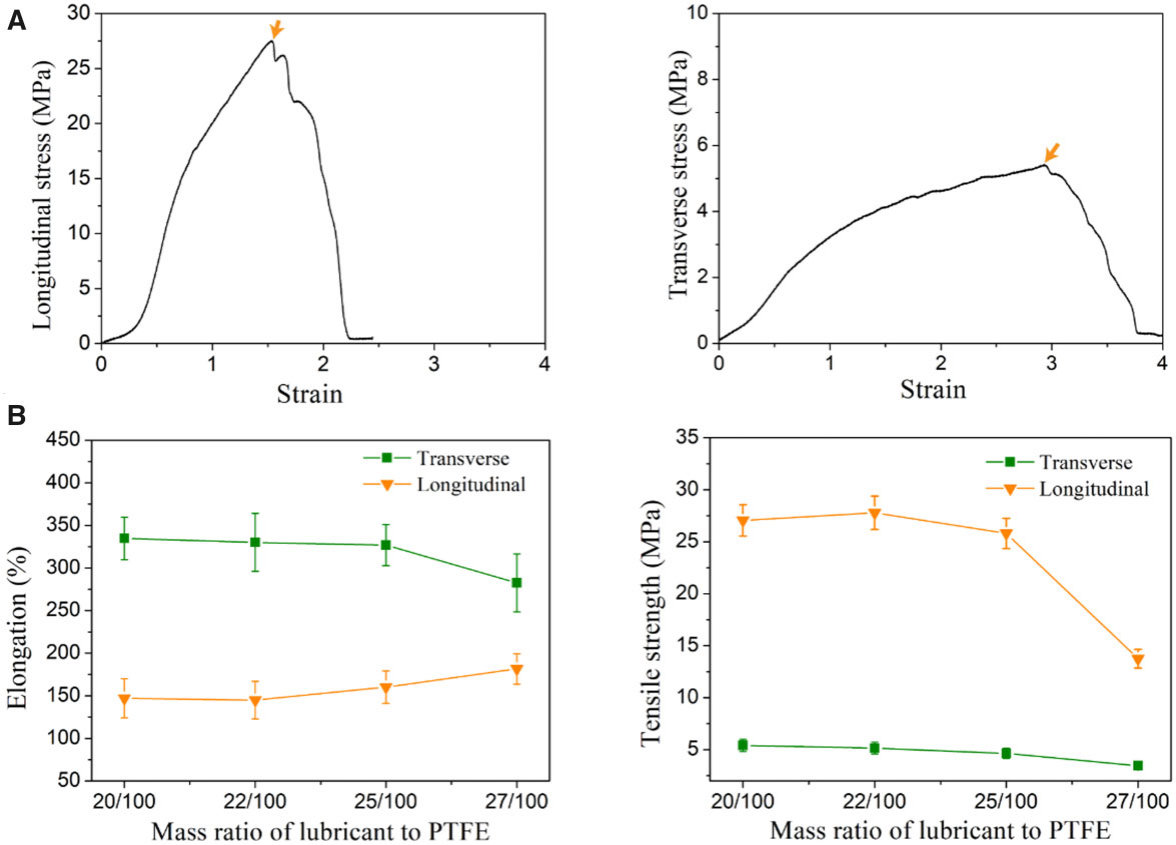
Effects of mass ratio of lubricant to PTFE powder on the ePTFE membrane. (**A**) Typical stress–strain curves of ePTFE membrane including longitudinal and transverse directions. The peak stress in the curve is defined as the tensile strength, and its corresponding strain as elongation. (**B**) The left and right are data of membrane elongation and strength.

In fabrication of ePTFE, a lubricant is required in the preforming and calendaring procedures to reduce the friction between PTFE particles and improve the processing performance [37]. We examined the effect of the lubricant amount on elongation and tensile strength of the ePTFE membranes in the transverse and longitudinal directions. In our study, when the mass ratio of lubricant to PTFE powder was lower than 20/100, the preformed billet was easy to break; while the ratio was higher than 27/100, it was easy to cause lubricant waste. Otherwise, PTFE and lubricants were well mixed. Even in such a wide feasible range of proportions to prepare ePTFE membranes, the mechanical properties of the as-fabricated ePTFE membranes exhibited some dependence on mass ratio of lubricant to PTFE powder, as indicated in Fig. 4. So, we have space to optimize the procedure parameters.

### Effects of longitudinal stretching ratio on the properties of ePTFE membranes

**Figure 5.**
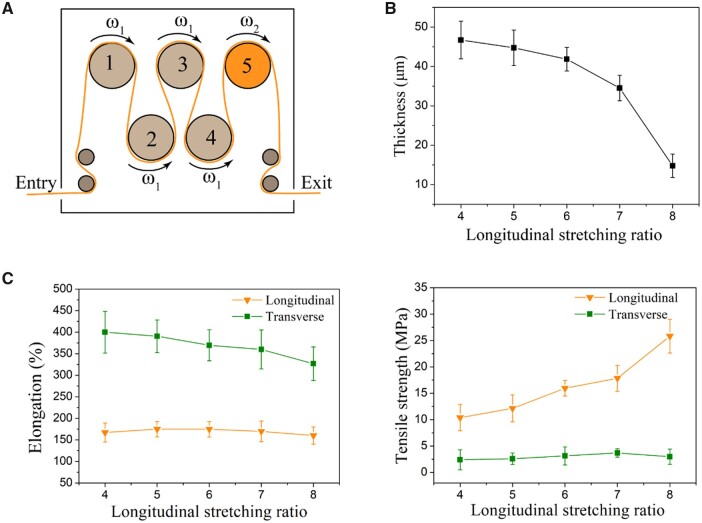
Effects of longitudinal stretching ratio on properties of ePTFE membrane. (**A**) Schematic diagram of longitudinal stretching of ePTFE membrane. The first four rollers had the same angular velocity denoted as ω_1_, while the last one had a larger velocity denoted as ω_2_. The longitudinal stretching ratio is defined as ω_2/_ω_1_. (**B**) Effect of longitudinal stretching ratio on membrane thickness. (**C**) Effect of longitudinal stretching ratio on ePTFE membrane elongation and strength.

In the longitudinal stretching, the primary PTFE tape containing the lubricant was processed by removing oil under heating conditions, and then stretched to form the ePTFE tape. Figure 5 shows the effects of the longitudinal stretching ratio on the performance of the ePTFE membranes. With the increase of the longitudinal stretching ratio, the thickness gradually decreased to ∼15 μm in our experiments. The transverse elongation rate decreased with the increase of stretching ratio, while the longitudinal elongation rate remained stable at ∼170%. The longitudinal tensile strength increased rapidly with longitudinal stretching ratio, and the transverse tensile strength fluctuated slightly within a small range of 2.4–3.7 MPa.

In our study, when the longitudinal stretching ratio was lower than 4, the membrane thickness was often uneven during the transverse stretching. When the ratio was greater than 8, the tape might be broken and the membrane was not suitable for transverse stretching due to overly high longitudinal fibril density.

### Effects of transverse stretching ratio on the properties of ePTFE membranes

**Figure 6.**
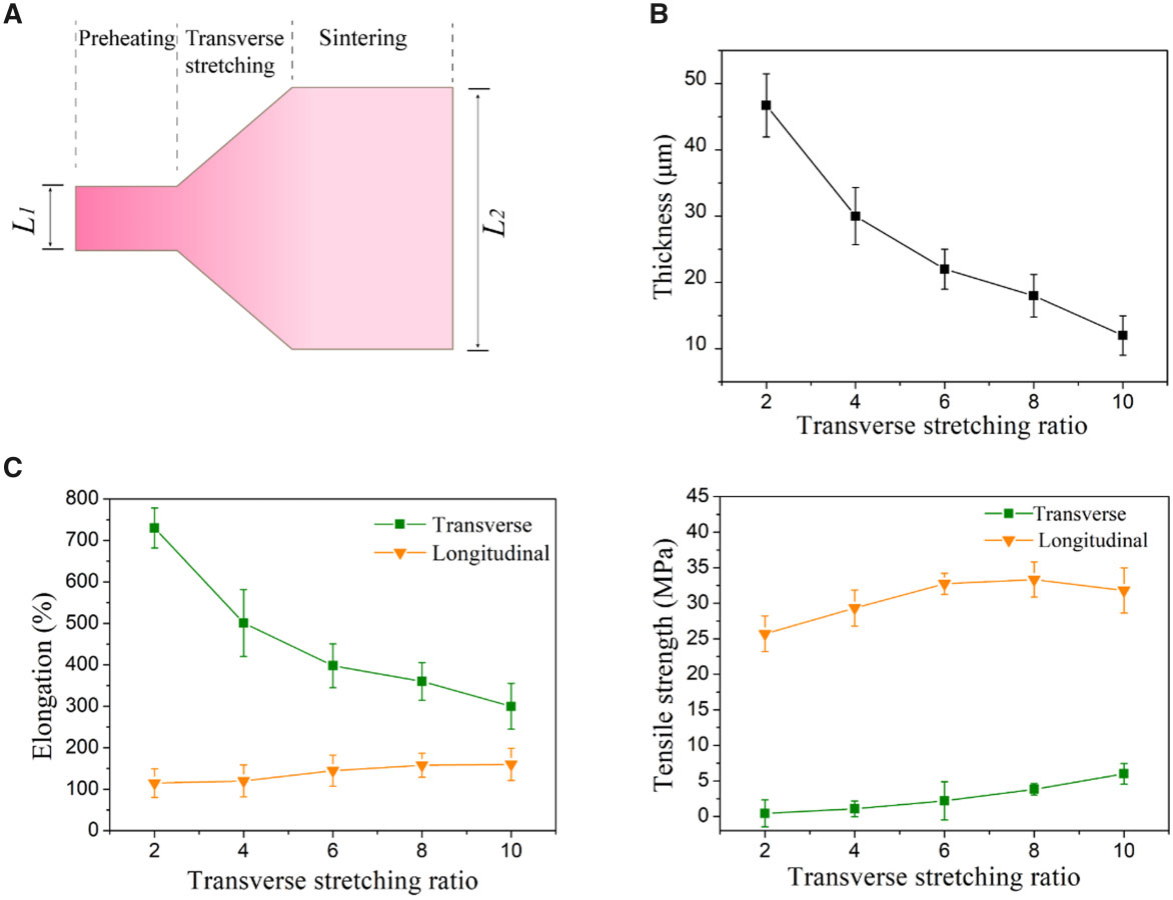
Effects of transverse stretching ratio on properties of ePTFE membrane. (**A**) Schematic diagram of transverse stretching of ePTFE membrane. The transverse stretching ratio is defined as *L*_2/_*L*_1_. Because of the limitation of the machine space, the maximum value of *L*_2/_*L*_1_ was 10. According to our experience, when the transverse stretching ratio was <2, an even membrane could not be obtained, and thus the transverse stretching ratio was between 2 and 10. (**B**) Effect of transverse stretching ratio on membrane thickness. (**C**) Effect of transverse stretching ratio on membrane elongation and strength.

The longitudinal stretching was followed by the transverse stretching. Figure 6 shows thickness, transverse and longitudinal elongation and tensile strength of the ePTFE membranes as a function of transverse stretching ratio. With the increase of the transverse stretching ratio, the thickness of the ePTFE membranes gradually decreased to ∼12 μm. The transverse tensile strength increased with the transverse stretching ratio, and meanwhile the longitudinal tensile strength changed slightly.

### Effects of sintering temperature on the structure and properties of ePTFE membranes

**Figure 7.**
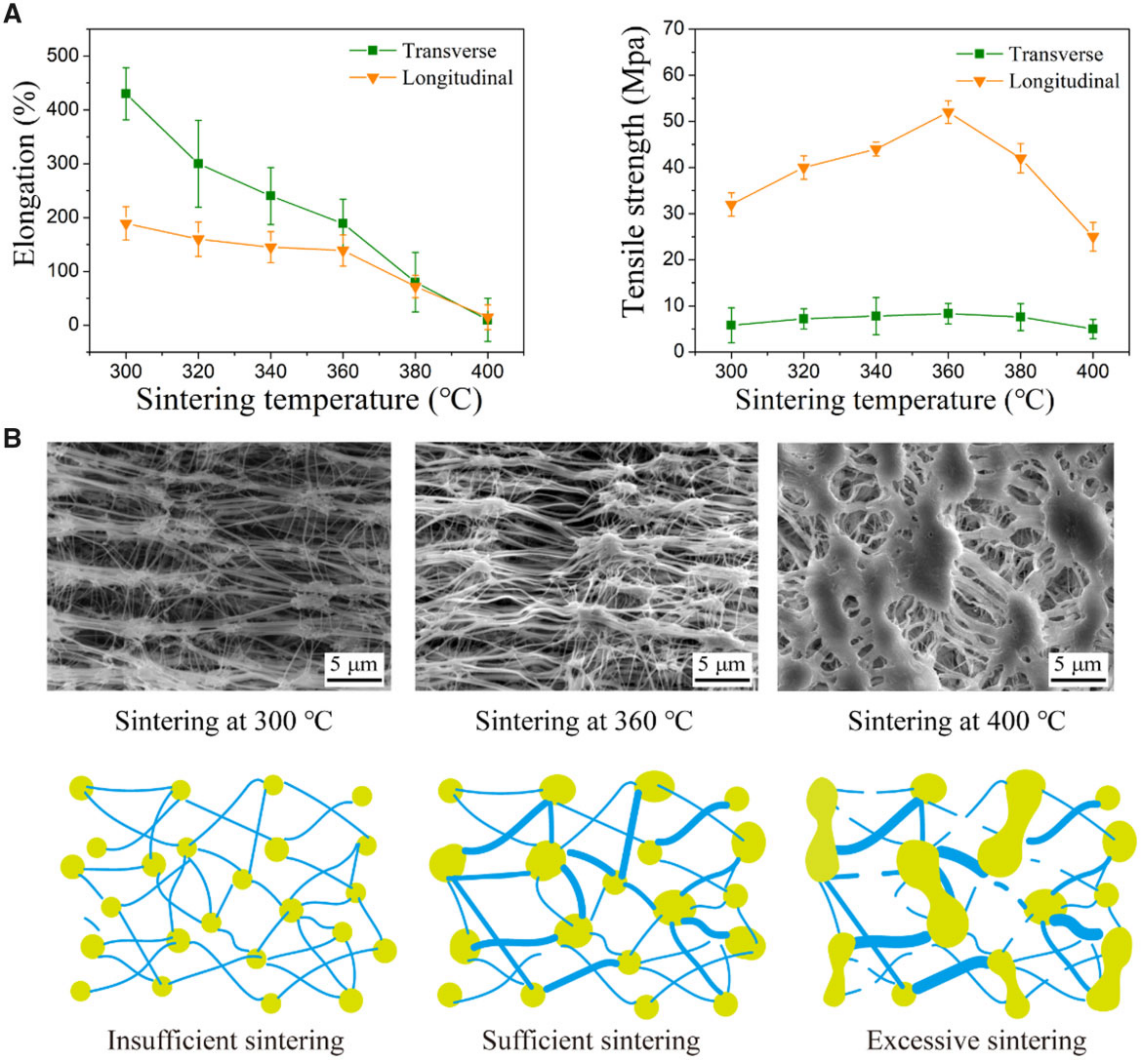
Effects of sintering temperature on properties of ePTFE membrane. (**A**) Membrane elongation and strength as a function of sintering temperature. With the increase of sintering temperature, both transverse and longitudinal elongations decrease. The longitudinal and transverse tensile strength get the highest values at the sintering temperature of 360°C. (**B**) SEM images of ePTFE membrane sintered at the indicated temperatures, and the corresponding schematic illustration of insufficient, sufficient and excessive sintering.

Sintering of the ePTFE membrane was carried out under certain stress and temperature after stretching, making the dispersed resin particles fuse with each other to some extent. During this process, parts of the polymer molecules change from the crystalline state to the amorphous state. After cooling, polymer molecules changed from amorphous to crystalline state, which kept the network structure of the ePTFE membrane and improved its tensile strength [42, 43]. As shown in Fig. 7A, with the increase of sintering temperature, the transverse and longitudinal elongations of our ePTFE membrane gradually decreased from 430 ± 48% and 189 ± 31%, respectively, and finally both decreased to around 75% at 400°C. The transverse and longitudinal tensile strengths of the ePTFE membrane increased first and then decreased with the increase of sintering temperature. The values at 360°C reached the maximum of 8.3 ± 0.2 and 52.0 ± 2.4 MPa, respectively.

In particular, we carried out SEM observations of the microstructure of the ePTFE membrane as-fabricated in industrial scale using our self-built facilities. The characteristic ‘node-fibril’ structure of ePTFE was observed in Fig. 7B. The sintering temperature was found to be critical for the microstructure formation of ePTFE, with typical cases of insufficient, sufficient or excessive sintering, demonstrated in Fig. 7B. Sintering at a low temperature such as below 300°C led to no sufficient strong fibril formation. Then, the ‘node-fibril’ structure could not be fixed, and the membrane was easy to retract when unwinding from the mandrel, resulting in lower tensile strength. With the increase of sintering temperature, the number of fine fibers decreased and the thick fibers were observed, especially the longitudinal thick fibers, which is conducive to the improvement of longitudinal tensile strength. So, the optimal tensile strength was achieved upon sintering at 360°C. Further increased sintering temperature showed a negative correlation to the tensile strength of the ePTFE membrane. As demonstrated by the SEM image of the specimen sintered at 400°C, the small fibers were significantly reduced, the adhesion occurred between the junctions and the original fiber structure was destroyed, which ultimately led to the decrease of flexibility and tensile strength of the ePTFE membrane.

The analysis of node-fiber structure of ePTFE membranes sintered at the three temperatures was made using ImageJ software. The results in Supplementary Fig. S2 strengthens our basic conclusion that an appropriate sintering temperature leads to the optimal ‘node-fibril’ structure.

### Thermal properties of ePTFE membranes

The crystallization and thermodynamic properties of the material were analyzed with differential scanning calorimetry. The typical curves are shown in Supplementary Fig. S3. At a heating rate of 10°C/min, melting transition peaks occurred at 348°C and 342°C for the manufactured ePTFE membrane and the PTFE raw material, respectively.

The degrees of crystallinity χ_c_ can be calculated from the equation χ_c_ = Δ*H*_m_/Δ*H*_m0_. Δ*H*_m0_ denotes the enthalpy of melting of fully crystalline PTFE and is equal to 71 J/g [44]. The calculated crystallinity of the membrane was 76%, which was slightly higher than that of the counterpart from the market (Supplementary Fig. S4), and that of the PTFE raw material was 99%. Because the crystallization property of ePTFE is very sensitive to temperature, there was no thermal history treatment process prior to the testing.

### Cytotoxicity test

**Figure 8.**
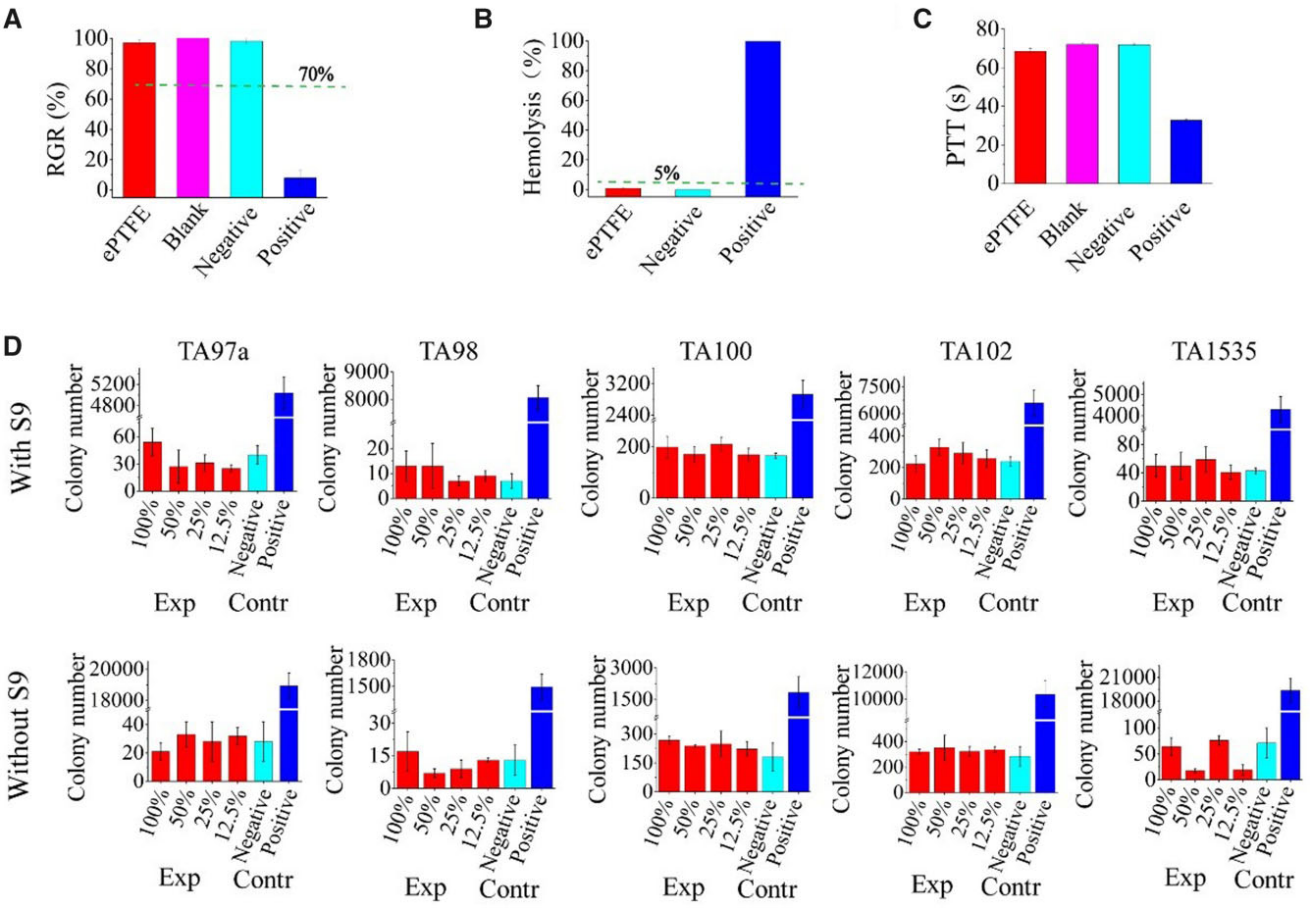
*In vitro* biocompatibility test and bacterial reverse mutation tests of sintered ePTFE membranes with five test strains (*Salmonella typhimurium* TA97a, TA98, TA100, TA102 and TA1535). (**A**) Cytotoxicity test of the sintered ePTFE membrane. SDS, HDPE and culture media are set as the positive, negative and blank controls, respectively. The negative control sample, positive control sample, blank control and sintered ePTFE membrane were extracted under the same extraction conditions (37°C, 24 h). The dashed line indicates the criteria of the corresponding ISO 10993.5 of *in vitro* cell viability for a medical device. (**B**) Hemolysis tests of the sintered ePTFE membrane. Normal saline and distilled water were used as negative and positive controls, respectively. The dashed line indicates the criteria of acceptable hemolysis for a medical device. (**C**) Coagulation test of the sintered ePTFE membrane. The negative group was HDPE, which does not activate coagulation. Glass beads were used as positive material with a shortened PTT. The blank group was untreated platelet plasma that provides a normal background PTT in the coagulation study. (**D**) Bacterial reverse mutation tests on the sintered ePTFE membrane. The DMSO extract from sintered ePTFE membrane was used as the experimental group, DMSO as the negative control group and the mutagen as the positive control group. Among them, 100% refers to the extract stock solution, 50%, 25%, 12.5% refers to the gradient dilution of the extract stock solution.

Biocompatibility is a basic requirement for any biomaterial [45–48], and cell-material interactions have been comprehensively investigated [49–55]. In this study, the cytotoxicity of the extract of our sintered ePTFE membranes was evaluated, and the results are shown in Fig. 8A. The sintered ePTFE membrane group showed up to 97 ± 2% relative cell viability after 72 h of culture, which was much higher than the corresponding ISO standard 10993.5 *in vitro* cell viability of medical devices by 70%, indicating that the material has good cytocompatibility and can afford a microenvironment for cell growth.

### Blood compatibility

Blood compatibility evaluation is an important part of verifying the biocompatibility of materials. In this study, two important *in vitro* blood compatibility indexes of the extracts of sintered ePTFE membrane, including hemolytic performance and anticoagulant property were evaluated. The results are shown in Fig. 8B and C. The hemolysis rate of the sintered ePTFE membrane read only 0.86 ± 0.3%, far lower than 5% stipulated by the corresponding ISO standard 10993.4 of hemolysis for the medical device, indicating that the sintered ePTFE membrane does not cause hemolysis.

We measured the time for a citrated plasma exposed to sample materials to form a clot when exposed to a suspension of phospholipid particles and calcium chloride. In this coagulation test, the test article is the activator. Material samples that show a shortened partial thromboplastin time (PTT) are activators of the intrinsic coagulation pathway. In our experiments, the sintered ePTFE membrane and control samples were incubated with plasma at 37°C for 15 min, and at the end of the incubation, the plasma was removed and tested using a coagulation analyzer. While the negative control group and platelet poor plasma group (blank group) resulted in PTT of 71.9 ± 0.6 s and 72.1 ± 0.6 s, respectively, the experimental group with sintered ePTFE membrane had PTT of 68.5 ± 1.5 s, which was close to that of the negative group and the blank group, indicating that the sintered ePTFE membrane did not lead to coagulation.

We also observed blood directly contacted with the membrane via thrombosis tests. According to the criteria of thrombosis, thrombosis was classified into five scoring grades: no or very trace thrombosis was assigned a score of 0; 1–25% of the total area/length of material covered by the formed thrombus was scored as 1; The formed thrombus covered 26–50% of the total area/length of the material as two points; 51–75% of the total area/length of material covered by the formed thrombus was score 3; the formed thrombus covered 76–100% of the total area/length of the material on a four-point scale. The thrombosis formation scores of the experimental group (the sintered ePTFE membranes made from our manufactured ePTFE) and the control group (the sintered ePTFE membrane made from purchased ePTFE) in the two animals are 1, 1 and 0, 1 respectively, as shown in Supplementary Table S1. Supplementary Figure S5 show the SEM images of the experimental group and the control group. Although a small number of platelets adhered to the surface at 1000 magnification, no obvious thrombus was formed which met the requirements of ISO 10993-4: 2017.

### Bacterial reverse mutation tests

The histidine auxotrophic strain of *Salmonella typhimurium* is unable to synthesize histidine, so the bacteria are essentially unable to grow on any medium lacking histidine. In the presence of a mutagen, the auxotrophic bacteria probably undergoes a reverse mutation in synthesizing histidine on their own, and thus grows and forms colonies. This principle can be employed to examine whether the test substance is a mutagen. Certain chemicals need to be metabolically activated to be mutagenic, and the addition of mammalian microsomal enzymes such as hepatic S9 fraction to the test system may compensate for the lack of a metabolically activated system in the *in vitro* assay [56].

We performed bacterial reverse mutation experiments (Ames experiments) on sintered ePTFE membranes. *Salmonella typhimurium* (MOLTOX) TA97a, TA98, TA100, TA102 and TA1535 were used to test the DMSO extracts of each dosage sample. Under the conditions of with S9 activation system and without S9 activation system, the results meet the acceptance criteria that the number of revertant mutant colonies of each strain should be no more than two times of the negative control group (DMSO), and the number of revertant mutant colonies of the positive control group strains should at least be three times of the negative control group. The test results are shown in Fig. 8D. The experimental results for the five strains were negative, and sintered ePTFE membranes were judged to be free of mutagenicity according to the requirements of ISO 10993.3 and ISO/TR 10993.33.

### Intracutaneous reactivity test

**Figure 9.**
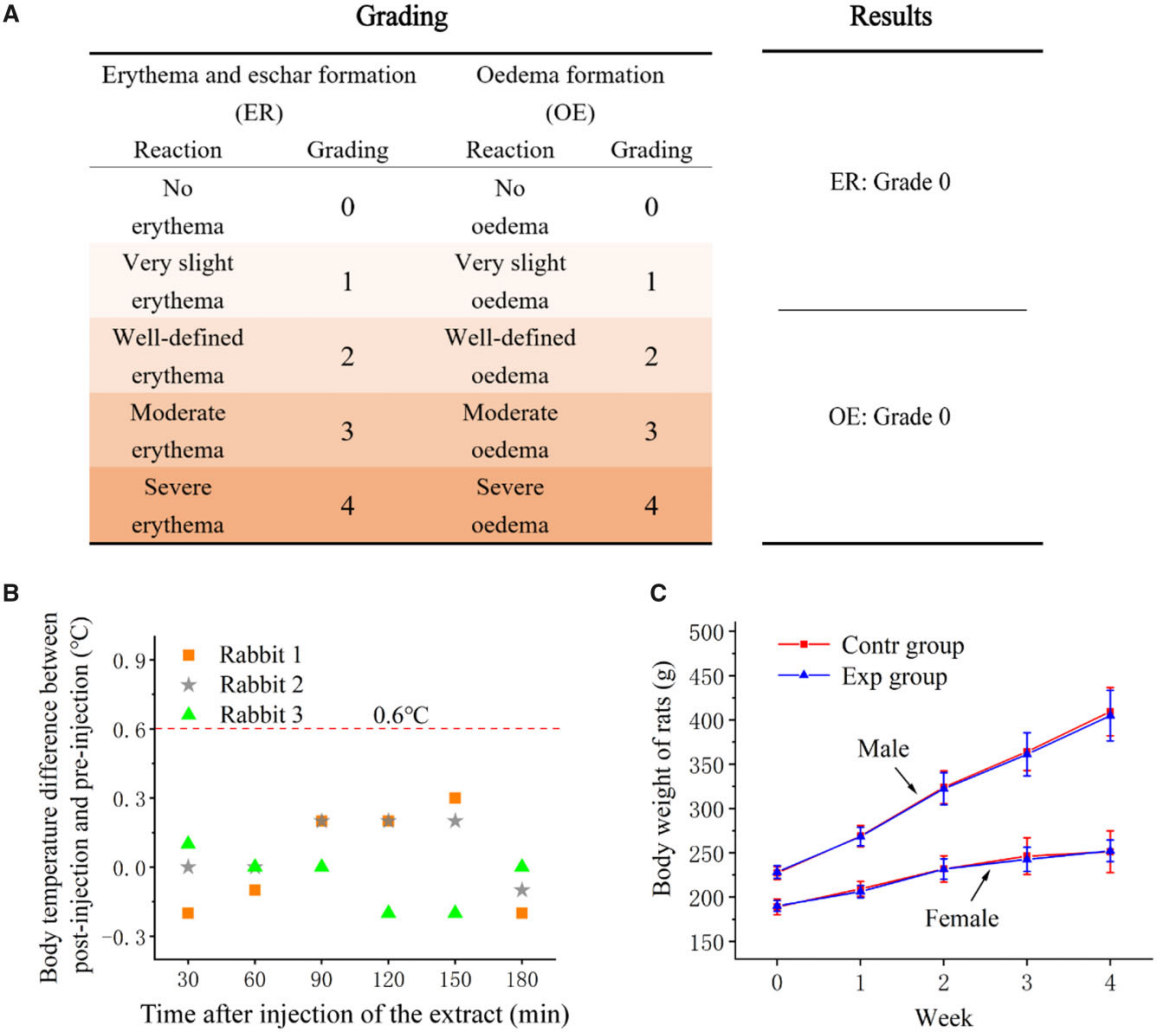
(**A**) Grading and results of intracutaneous reactivity test of the sintered ePTFE membrane. (**B**) Results of pyrogen test with sintered ePTFE membrane. Difference in body temperature on three rabbits after and before injection of the extract within 3 h. (**C**) Results of subchronic systemic toxicity test with sintered ePTFE membrane. Body weight variations of the experimental male and female rats in subchronic systemic toxicity test within 4 weeks. The extracts of sintered ePTFE membrane and normal saline were the experimental group and the control group, respectively.

According to the evaluation criteria of intracutaneous reactivity experimental results, two phenomena of intracutaneous reactivity in animals, erythema and edema, can be divided into 0–4 points (Supplementary Table S2). No erythema and edema were observed in the sample group and control group (blank extract solvent without sample) during the observation period of animal experiment (Supplementary Table S3). The average scores of sintered ePTFE membrane in both extract solutions and both solvent controls was 0 (Fig. 9A), which indicated that sintered ePTFE membrane did not induce irritation to the tissue.

### Pyrogen test

Pyrogen experiments for sintered ePTFE membranes were performed with home immunity. During the test period, the increase of body temperature in all of the three experimental animals was <0.6°C, and the sum of the body temperature increases was no more than 1.3°C, as shown in Supplementary Table S4 and Fig. 9B, meeting the standard requirements of the pyrogen experiment ISO 10993-11: 2017, indicating that the sintered ePTFE membrane had no thermogenic effect.

### Subchronic systemic toxicity test

Throughout the 28-day experiment, no obvious abnormal performance occurred in either test or control animals, and the body weights of the rats all grew normally overtime during the rearing period (Fig. 9C).

Hematological tests were carried out concerning white blood cell, red blood cell, hemoglobin, hematocrit, platelet, neutrophils, lymphocytes, monocytes, eosinophils, basophils, prothrombin time, activated PTT. The experimental groups and the control groups were within the normal range and with no statistically significant difference, as shown in Supplementary Tables S5 and S6 for female and male rats, respectively.

Blood biochemistry was also tested including alanine aminotransferase, aspartate aminotransferase, alkaline phosphatase, γ-glutamyl transpeptidase, total bilirubin, total protein, albumin, glucose, creatinine, urea, cholesterol, triglyceride, inorganic phosphorus, Ga^2+^, Na^+^, K^+^, Cl^−^. The experimental groups and the control groups were within the normal range and with no statistically significant difference, as shown in Supplementary Tables S7 and S8 for female and male rats, respectively.

The relative weights of each organ were normal including brain, thymus, heart, liver, spleen, adrenal, kidney, ovary, uterus, testis and epididymis. There was no statistical difference between the experimental and control groups, as shown in Supplementary Tables S9 and S10 for female and male rats, respectively.

### Preliminary *in vivo* biocompatibility of sintered ePTFE membranes

**Figure 10.**
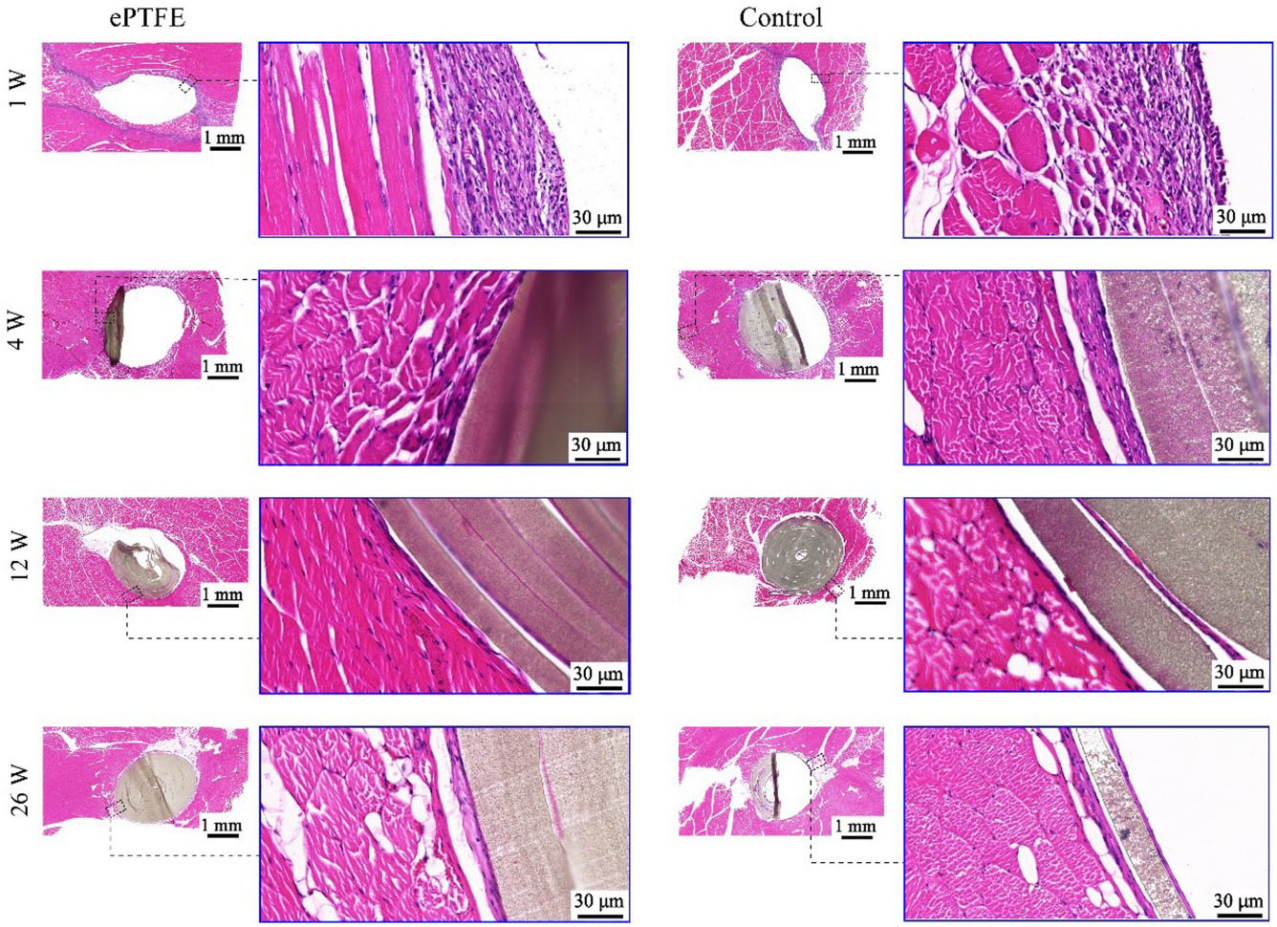
Optical micrographs of HE-stained slices of the implanting sites. Our manufactured sintered ePTFE membrane and control group were implanted in the muscle of the rabbits for the indicated weeks prior to the sampling.

We implanted sintered ePTFE membranes into the dorsal muscles of rabbits to access their immunological responses. The typical HE-stained images are presented in Fig. 10. After 1 week, the pathological results showed that fibrous capsules were formed around the implants with scattered inflammatory cells (Supplementary Fig. S6). And there was no significant difference of local tissue inflammatory response between the experimental group and the control group, which was a legally marketed and clinically acceptable sintered ePTFE membrane from the market. Fibrous capsules gradually became thinner and the inflammatory cells decreased around the implants 4 weeks after implantation. There was no significant difference of tissue response between the experimental group and the control group. At Week 12 after implantation, thin fibrous capsules were formed around the implants, and inflammatory cells were almost invisible. At Week 26 after implantation, the interface between implant/tissue of the test sample group exhibited a thin layer of capsule.

## Discussion

With the improvement of quality of life and the coming of the aging society, the demand for biomedical materials continues to rise, especially in the treatment of cardiovascular diseases, orthopedics diseases, plastic surgery etc. [57–62]. While much progress has been made in various medical devices and biomaterials [48, 63–68], the available medical-grade raw materials including some specific physical forms are limited [69–72]. Owing to the membrane’s physical form and the excellent mechanical properties, ePTFE has a unique advantage in the benign fit of human tissue, and has been used in biomedical implants [14]. Nowadays more and more research has been made to develop high-performance biomaterials [73–77]. In the present study, we have improved the processing to fabricate medical-grade ePTFE material with uniform membrane thickness, good mechanical and biological properties.

Biaxial stretching adopts a longitudinal stretching followed by a transverse stretching. In the transverse stretching, both sides of an ePTFE tape are clamped on and move with the track. However, a large longitudinal retraction force is caused in the longitudinal stretching process, and the longitudinal retraction tendency is further aggravated in the transverse stretching process with the extension of the transverse width. This leads to a ‘bowing effect’ at the ends of a membrane. Such an effect is manifested as the uneven membrane thickness, leading to a membrane thicker in the middle and thinner on both sides. To solve this problem, we have designed an olive-shaped winding roller. With this roller, the middle of the tape has a larger longitudinal stretching range compared with the two sides when stretching longitudinally. Therefore, the extra stretching amplitude in the middle relative to both sides of the longitudinal stretching can make up for the longitudinal retraction caused by the ‘bowing effect’, which is conducive to the formation of uniform membrane thickness. Our pre-experiment illustrates that the appropriate middle diameter (*d*_2_) is 200 mm, the side diameter (*d*_1_) is 150 mm and the angle (α) between the roller surface and the axis is 30°. The larger size of the olive roller will be limited by the whole equipment, in particular the part in the winding process. Figure 3 confirms that the ePTFE membranes prepared by the olive roller winding process have a more uniform membrane thickness than the common winding roller.

In the study, we have also found that the addition of lubricants is conducive to improving the performance of the ePTFE membrane, but overly more lubricants lead to the decline of the tensile strength of the ePTFE membrane (Fig. 4). It seems that the excessive amount of lubricant added causes the over-dispersion of PTFE particles, which reduces the friction between PTFE particles in the subsequent extrusion, calendaring and other processes, resulting in the reduction of fibers drawn between particles and the decrease of tensile strength.

We have carefully examined the effects of longitudinal stretching ratio on the transverse tensile strength. An optimal ratio was found, as shown in Fig. 5. When the longitudinal stretching ratio is overly high, there are too many fibers oriented along the longitudinal direction, and the ePTFE tape becomes thinner. At this time, when the transverse stretching is performed, relatively fewer fibers are available for the transverse orientation, so the membrane becomes thinner and the transverse tensile strength decreases. In addition, we found that the influence of the transverse stretching ratio on the longitudinal tensile strength is 2-fold (Fig. 6). The transverse tensile force promotes the formation of fibers in the 3D network and improves the overall strength of the membrane, which is beneficial to the longitudinal strength. However, when the transverse stretching reaches a certain extent, the longitudinal fibers are gradually consumed by the formation of branching fibers in other directions, resulting in fewer fibers along the longitudinal direction and finer fibers. This is an adverse side to the longitudinal strength, which may interpret why the longitudinal tensile strength increases first and then decreases slightly with the transverse stretching.

Compared with the raw PTFE resin prior to stretching, the ePTFE membrane after biaxial stretching has reduced crystallinity and expanded amorphous area (Supplementary Fig. S3). It might come from the destruction of some crystalline areas, the sliding of lamellar crystals and molecular chains. The unstretched crystalline region becomes a node, which is connected by several fibrils. At this time, a network microporous structure is formed, and the toughness and strength of ePTFE membrane are improved.

In the process of biaxial stretching of ePTFE membrane, the ambient temperature is controlled far below its melting point of 327°C, but the lower temperature does not make the stretched ePTFE membrane microporous structure well preserved, making the dimensional stability of the finished product poor. Therefore, further sintering treatment is required. With the increase of sintering temperature, the nodes of ePTFE film become larger and larger. The higher the temperature, the higher the proportion of crystalline zone transformed into amorphous zone. More molten PTFE particles and fibrils diffuse away and probably fuse with each other, resulting in larger nodes and stronger fibrils formation, thus improving the strength of the membrane. However, over-sintering also leads to the reduction of the uniformity of pore size, the uneven thickness of the fibrils, and the partial fibrils may break under high temperature, leading to decreased elongation and breaking. The influence of sintering temperature on the microstructure and properties of ePTFE membranes is well demonstrated in Fig. 7. As a medical material, the high porosity of ePTFE might be beneficial for cell adhesion and growth. In the applications of covered stents and artificial blood vessels, ePTFE requires high strength to prevent rupture caused by blood flow impact. The sintering temperature between 300°C and 340°C can well balance these two aspects is suitable.

A series of *in vitro* biocompatibility experiments and animal experiments is helpful to evaluate the biosafety of a material [78–81]. As can be seen from biological characterizations (Figs 8–10), sintered ePTFE membranes met the ISO 10993 series of standards for biological evaluation of medical devices and did not show the phenomena of accelerated coagulation, thermal effect, nor sensitization and causing intradermal irritation. In addition, no abnormalities were found in the contrast of hematological indices, blood biochemical indices or organ coefficients between experimental and control animals, fully illustrating the safety of this sintered ePTFE membrane at the implant level. Histopathological examination of the animals also showed that the sintered ePTFE membrane did not induce excessive inflammatory reactions and could fit well with the muscle tissue after implantation *in vivo*, further demonstrating its biocompatibility. We expect that the medical-grade ePTFE membrane fabricated in industrial scale in this study can be applied as the raw material of advanced medical devices, following our previous clinical study of stent-graft for interventional treatment of aortic dissection [41].

## Conclusion

This study has designed an olive-shaped winding roller to improve the biaxial stretching in fabrication of ePTFE membrane from PTFE resin. Confirmed with a series of physical and biological characterizations, a medical-grade ePTFE membrane with uniform membrane thickness, excellent mechanical properties and good biocompatibility has been successfully prepared. Biaxial stretching of PTFE led to well controlled node-fibril microstructure, and the medical ePTFE membrane was fabricated with self-built apparatus in industrial scale. In the long run, the ePTFE membrane with excellent performance can be applied not only in implanted stents, but also in artificial blood vessels, biological patches, electronic equipment and even aerospace and other fields to fully realize its added value.

## Supplementary Material

rbad056_Supplementary_Data
