# Supplementary material for: Biaxial stretching of polytetrafluoroethylene in industrial scale to fabricate medical ePTFE membrane with node-fibril microstructure
Source: Regen Biomater. 2023 Jun 2;10:rbad056. doi: 10.1093/rb/rbad056 (PMC10310521; doi:10.1093/rb/rbad056)
Supplement: rbad056_Supplementary_Data [file rbad056_supplementary_data.docx]

**Supporting Information**

**Biaxial stretching of polytetrafluoroethylene in industrial scale to fabricate medical ePTFE membrane with node-fibril microstructure**

Gang Wang^1,2,3^, Yusheng Feng^2^, Caiyun Gao^1^, Xu Zhang^2,3^, Qunsong Wang^1^, Jie Zhang^2,3^, Hongjie Zhang^1^, Yongqiang Wu^2,3^, Xin Li^1^, Lin Wang^2,3^, Ye Fu^1^, Xiaoye Yu^1^, Deyuan Zhang^2^, Jianxiong Liu^2^, Jiandong Ding^1, *^

^1^State Key Laboratory of Molecular Engineering of Polymers, Department of Macromolecular Science, Fudan University, Shanghai 200438, China

^2^R&D Center, Lifetech Scientific (Shenzhen) Co., Ltd., Shenzhen 518057, China

^3^R&D Center, Lifevalve Medical Scientific Co., Ltd, Shenzhen 518057, China

* Corresponding author. Email: jdding1@fudan.edu.cn (J.D. Ding)

The supporting information presents supplementary methods and supplementary results with 6 supplementary figures and 10 supplementary tables shown in sequence mentioned in the main manuscript.

**Supplementary Methods**

**
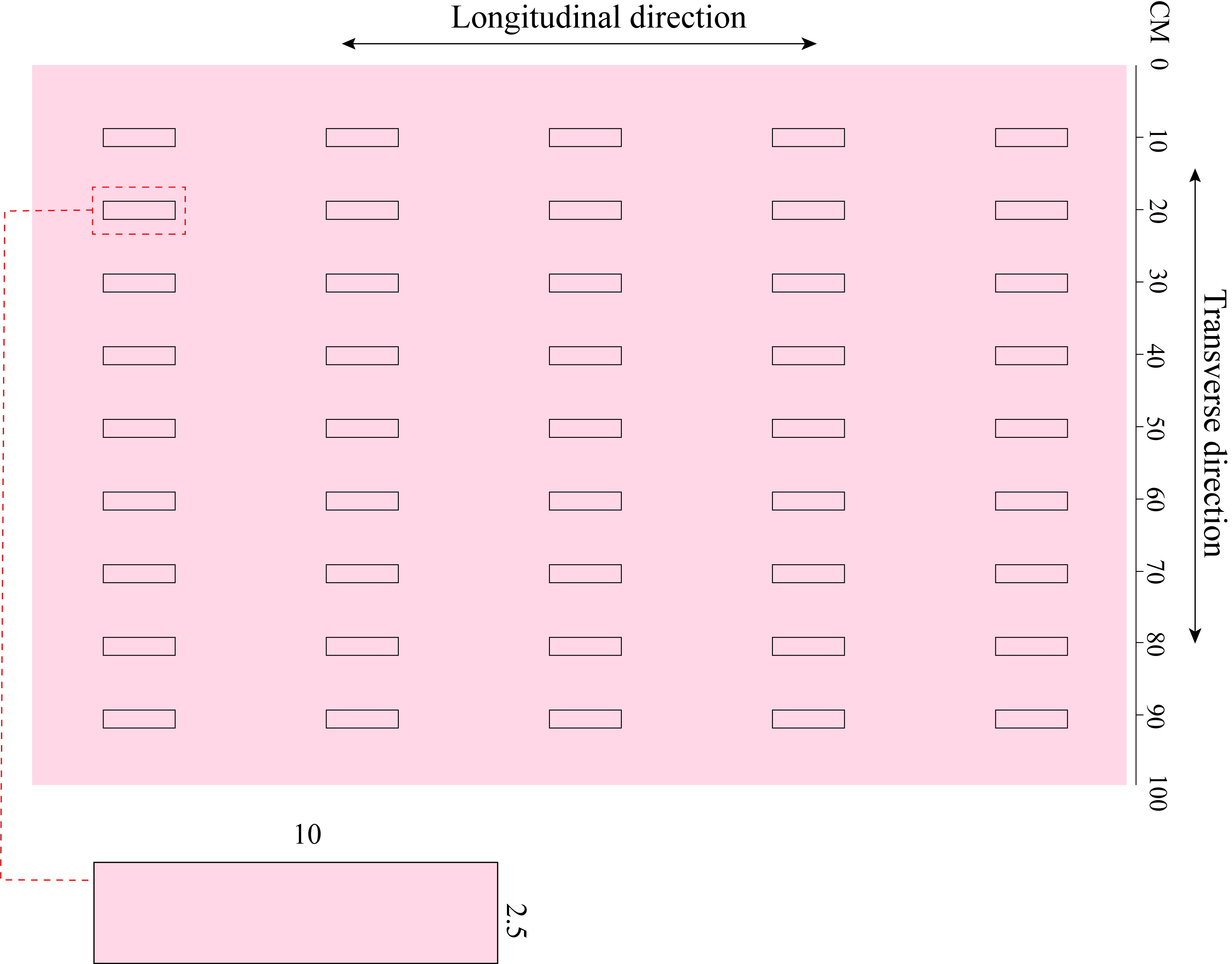
**

**Figure S1. Schematic diagram of sampling from an ePTFE membrane fabricated by biaxial stretching of PFTE resin.** Nine sites were sampled in the transverse direction with a span of 100 cm. Take 5 sites for statistics along the longitudinal direction in the same transverse position.

**Supplementary Results**


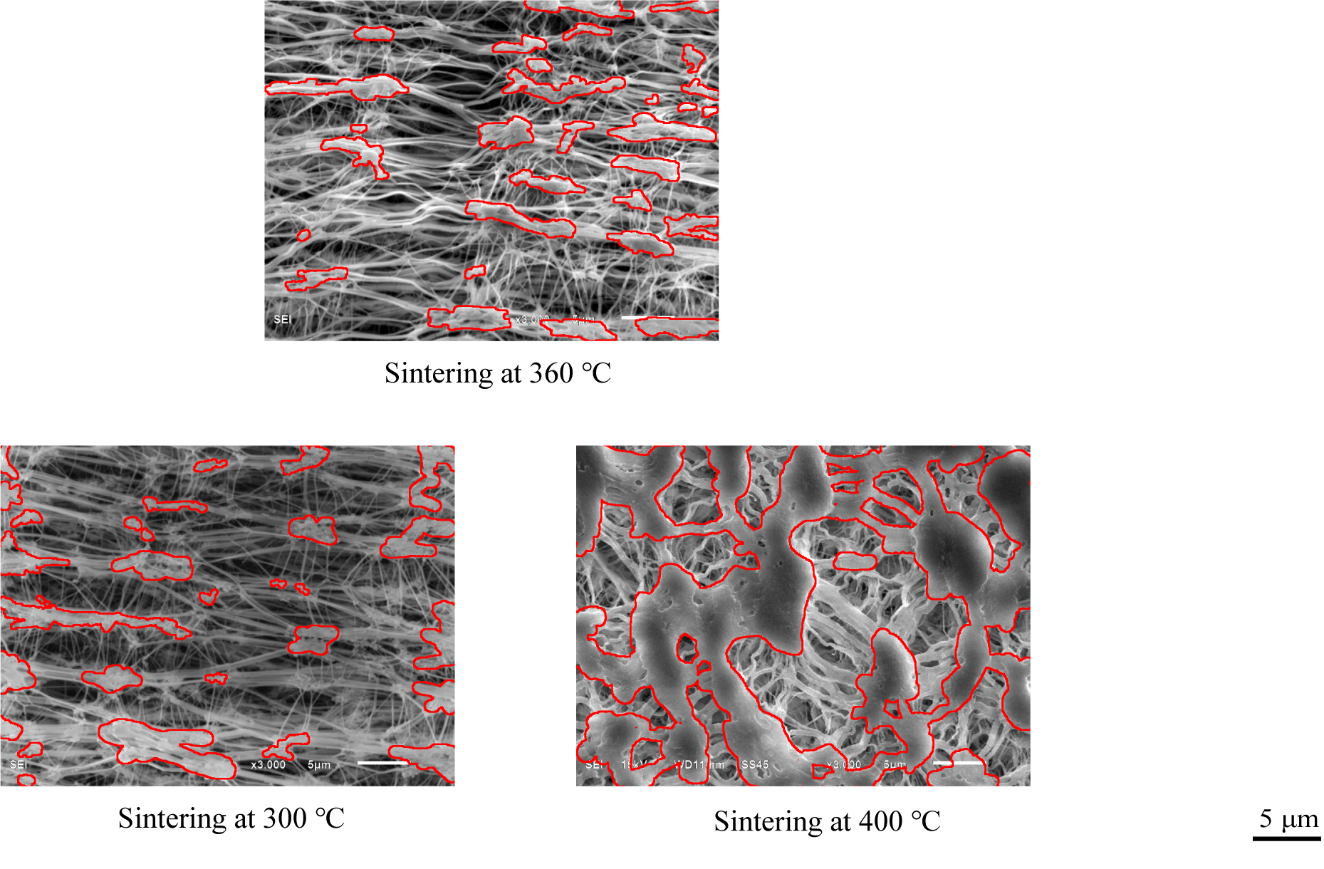


**Figure S2.** **Schematic diagram of node-fiber structure analysis of ePTFE membranes sintered at three temperatures using ImageJ software.** The part surrounded by the red lines in the figure is the node structure of ePTFE membrane. The cumulative area circled by the red lines in the left image accounts for less of the overall structure, only 11.8%, and the nodes are relatively small. The nodes of the ePTFE membrane sintered at 360℃ slightly increase, accounting for 13.8% of the overall structure. When the sintering temperature is 400℃, the proportion of nodes in the overall structure of the ePTFE membrane increases significantly to 46%. The nodes are larger, but some fibers break, which may account for the decrease of mechanical strength.





**Figure S3. Differential scanning calorimetry (DSC) curves of our manufactured ePTFE membranes and the PTFE raw material.**





**Figure S4. DSC curve of a commercialized ePTFE membrane.** At a heating rate of 10°C/min，the commercialized ePTFE membrane exhibited melting transition peak at 348°C. Because the crystallization property of ePTFE is very sensitive to temperature, there was no thermal history prior to testing. The degrees of crystallinity χ_c_ of membrane can be calculated from the equation χ_c_ = Δ*H*_m_ / Δ*H*_m0_, resulting in 63%. Δ*H*_m_ of the commercialized ePTFE membrane is 44.9 J/g and the pure PTFE is 71 J/g .

**Table S1. Result of thrombus formation test of the ePTFE membrane**

| Groups | Scores of thrombus formation | |
| --- | --- | --- |
|  | Animal 1 | Animal 2 |
| Experimental group | 0 | 1 |
| Control group | 1 | 1 |


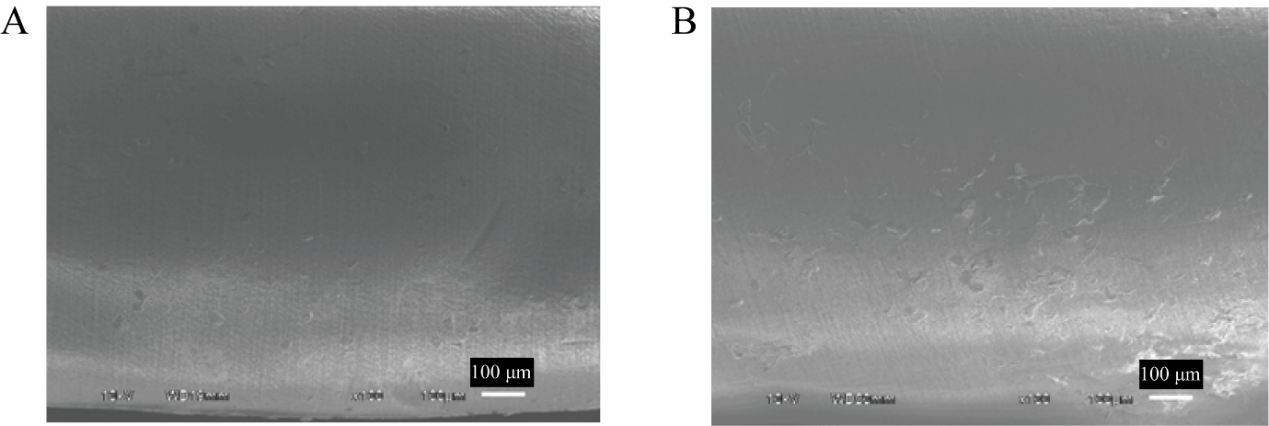


**Figure S5. SEM images of thrombosis testing with (A) manufactured sintered ePTFE membrane (experimental group) and (B) purchased ePTFE membrane (control group).** The thrombosis test followed the protocol recommended by ISO 10993-4: 2017: Biological evaluation of medical devices—Part 4: Selection of tests for interactions with blood. There was minimal to nonexistent thrombosis checked by the SEM image. According to ISO 10993-4: 2017, the thrombosis scores were Grade 0 in both groups.

**Table S2. Grading system for intracutaneous reactivity test**

| Erythema and eschar formation (ER) | | Oedema formation (OE) | |
| --- | --- | --- | --- |
| Reaction | Grading | Reaction | Grading |
| No erythema | 0 | No oedema | 0 |
| Very slight erythema (barely perceptible) | 1 | Very slight oedema (barely perceptible) | 1 |
| Well-defined erythema | 2 | Well-defined oedema (edges of area well-defined by definite raising) | 2 |
| Moderate erythema | 3 | Moderate oedema (raised approximately 1 mm) | 3 |
| Severe erythema (beet redness) to eschar formation preventing grading of erythema | 4 | Severe oedema (raised more than 1 mm, and extending beyond exposure area) | 4 |

**Table S3. The results of intracutaneous reactivity test**

| Groups | Animal NO. | 24h (ER/OE) | 48h (ER/OE) | 72h (ER/OE) | Score Average |
| --- | --- | --- | --- | --- | --- |
| ePTFE membrane with polar solvent | 1 | 0/0 | 0/0 | 0/0 | 0 |
|  | 2 | 0/0 | 0/0 | 0/0 | 0 |
|  | 3 | 0/0 | 0/0 | 0/0 | 0 |
| ePTFE membrane with non-polar solvent | 1 | 0/0 | 0/0 | 0/0 | 0 |
|  | 2 | 0/0 | 0/0 | 0/0 | 0 |
|  | 3 | 0/0 | 0/0 | 0/0 | 0 |
| Control group with polar solvent | 1 | 0/0 | 0/0 | 0/0 | 0 |
|  | 2 | 0/0 | 0/0 | 0/0 | 0 |
|  | 3 | 0/0 | 0/0 | 0/0 | 0 |
| Control group with non-polar solvent | 1 | 0/0 | 0/0 | 0/0 | 0 |
|  | 2 | 0/0 | 0/0 | 0/0 | 0 |
|  | 3 | 0/0 | 0/0 | 0/0 | 0 |

**Table S4. The results of pyrogen test**

| Animal  NO. | Baseline  Temperature (℃) | | | Temperature of every 30 min intervals (℃) | | | | | | Maximum temperature rise (℃) |
| --- | --- | --- | --- | --- | --- | --- | --- | --- | --- | --- |
|  | T1 | T2 | T3 | 30 min | 60 min | 90 min | 120 min | 150 min | 180 min |  |
| 1 | 39.2 | 39.0 | 39.1 | 38.9 | 39.0 | 39.3 | 39.3 | 39.4 | 38.9 | 0.3 |
| 2 | 38.8 | 39.0 | 38.9 | 38.9 | 38.9 | 39.1 | 39.1 | 39.1 | 38.8 | 0.2 |
| 3 | 39.2 | 39.4 | 39.3 | 39.4 | 39.3 | 39.3 | 39.1 | 39.1 | 39.3 | 0.1 |
| Total rise in body temperature | | | | | | | | | | 0.6 |

**Table S5. The results of hematological tests in female rats (*n* = 10)**

| Parameters | Control（mean ± SD） | Exp（mean ± SD） | Statistical difference |
| --- | --- | --- | --- |
| WBC (10^9^/L) | 3.78 ± 1.12 | 3.09 ± 0.68 | None |
| RBC (10^12^/L) | 6.77 ± 0.32 | 7.24 ± 0.05 | None |
| Hgb (g/L) | 130.22 ± 7.26 | 138.20 ± 9.67 | None |
| Hct (%) | 37.54 ± 1.65 | 40.06 ± 2.57 | None |
| PLT (10^9^/L) | 995.89 ± 109.24 | 1025.20 ± 119.61 | None |
| NEUT (%) | 16.86 ± 5.96 | 13.16 ± 6.16 | None |
| LYMPH (%) | 74.76 ± 7.28 | 78.22 ± 6.34 | None |
| MONO (%) | 6.50 ± 1.27 | 7.03 ± 1.88 | None |
| EO (%) | 1.56 ± 0.62 | 1.28 ± 0.32 | None |
| BASO (%) | 0.33 ± 0.26 | 0.31 ± 0.19 | None |
| PT (s) | 7.54 ± 0.18 | 7.60 ± 0.12 | None |
| APTT (s) | 17.88 ± 1.31 | 16.97 ± 1.20 | None |

Note: “None” indicates no statistical difference between the two groups since *p* > 0.05.

- WBC: White blood cell;
- RBC: Red blood cell;
- Hgb: Hemoglobin;
- Hct: Hematocrit;
- PLT: Platelet;
- NEUT: Neutrophils proportion;
- LYMPH: Lymphocytes proportion;
- MONO: Monocytes proportion;
- EO: Eosinophils proportion;
- BASO: Basophils proportion;
- PT: Prothrombin time;
- APTT: Activated partial thromboplastin time.

**Table S6. The results of hematological tests in male rats (*n* = 10)**

| Parameters | Control（mean ± SD） | Exp（mean ± SD） | Statistical difference |
| --- | --- | --- | --- |
| WBC (10^9^/L) | 4.42 ± 1.37 | 4.39 ± 1.81 | None |
| RBC (10^12^/L) | 7.31 ± 0.28 | 7.31 ± 0.21 | None |
| Hgb (g/L) | 140.80 ± 5.05 | 138.20 ± 3.88 | None |
| Hct (%) | 40.57 ± 1.31 | 40.04 ± 1.02 | None |
| PLT (10^9^/L) | 1035.10 ± 85.15 | 998.90 ± 64.32 | None |
| NEUT (%) | 16.22 ± 5.08 | 13.58 ± 4.31 | None |
| LYMPH (%) | 74.68 ± 5.31 | 78.50 ± 5.65 | None |
| MONO (%) | 7.15 ± 0.87 | 6.28 ± 1.37 | None |
| EO (%) | 1.75 ± 0.51 | 1.39 ± 0.54 | None |
| BASO (%) | 0.20 ± 0.15 | 0.25 ± 0.17 | None |
| PT (s) | 8.85 ± 0.43 | 8.82 ± 0.45 | None |
| APTT (s) | 18.73 ± 1.43 | 18.18 ± 1.34 | None |

**Table S7. The results of clinical biochemical tests in female rats (*n* = 10)**

| Parameters | Control（mean ± SD） | Exp（mean ± SD） | Statistical difference |
| --- | --- | --- | --- |
| ALT (U/L) | 23.58 ± 3.32 | 27.94 ± 7.62 | None |
| AST (U/L) | 90.85 ± 16.12 | 100.02 ± 17.66 | None |
| ALP (U/L) | 81.67 ± 15.79 | 80.49 ± 22.48 | None |
| γ-GT (U/L) | 0.51 ± 0.22 | 0.71 ± 0.31 | None |
| TBIL (μmol/L) | 1.10 ± 0.46 | 1.01 ± 0.26 | None |
| TP (g/L) | 68.68 ± 6.06 | 67.58 ± 4.36 | None |
| ALB (g/L) | 48.68 ± 3.98 | 46.96 ± 3.73 | None |
| GLU (mmol/L) | 6.38 ± 0.47 | 6.46 ± 0.58 | None |
| CREA (μmol/L) | 32.50 ± 3.27 | 31.10 ± 1.91 | None |
| UREA (mmol/L) | 5.96 ± 1.00 | 5.95 ± 0.52 | None |
| CHOL (mmol/L) | 2.16 ± 0.40 | 2.05 ± 0.38 | None |
| TG (mmol/L) | 0.25 ± 0.10 | 0.21 ± 0.07 | None |
| Ga^2+^ (mmol/L) | 2.52 ± 0.07 | 2.49 ± 0.10 | None |
| IP (mmol/L) | 1.99 ± 0.18 | 2.00 ± 0.19 | None |
| Na^+^ (mmol/L) | 142.68 ± 0.57 | 143.39 ± 1.06 | None |
| K^+^ (mmol/L) | 3.96 ± 0.17 | 3.81 ± 0.27 | None |
| Cl^-^ (mmol/L) | 104.61 ± 1.25 | 105.19 ± 1.48 | None |

Note: “None” indicates no statistical difference between the two groups since *p* > 0.05.

- ALT: Alanine aminotransferase;
- AST: Aspartate aminotransferase;
- ALP: Alkaline phosphatase;
- γ-GT: γ-glutamyl transpeptidase;
- TBIL: Total bilirubin;
- TP: Total protein;
- ALB: Albumin;
- GLU: Glucose;
- CREA: Creatinine;
- UREA: Urea;
- CHOL: Cholesterol;
- TG: Triglyceride;
- Ca^2+^: Calcium ion;
- IP: Inorganic phosphorus;
- Na^+^: Sodium ion;
- K^+^: Potassium ion;
- Cl^-^: Chloride ion

**Table S8. The results of clinical biochemical tests in male rats (*n* = 10)**

| Parameters | Control（mean ± SD） | Exp（mean ± SD） | Statistical difference |
| --- | --- | --- | --- |
| ALT (U/L) | 33.93 ± 3.89 | 36.51 ± 5.98 | None |
| AST (U/L) | 140.95 ± 16.53 | 128.54 ± 23.66 | None |
| ALP (U/L) | 214.00 ± 33.74 | 212.42 ± 23.90 | None |
| γ-GT (U/L) | 0.53 ± 0.14 | 0.72 ± 0.15 | None |
| TBIL (μmol/L) | 0.93 ± 0.24 | 0.87 ± 0.24 | None |
| TP (g/L) | 53.03 ± 1.39 | 52.40 ± 2.81 | None |
| ALB (g/L) | 34.05 ± 0.83 | 34.12 ± 1.30 | None |
| GLU (mmol/L) | 6.16 ± 0.46 | 6.53 ± 0.49 | None |
| CREA (μmol/L) | 27.20 ± 3.16 | 26.40 ± 3.50 | None |
| UREA (mmol/L) | 5.44 ± 0.58 | 5.49 ± 0.84 | None |
| CHOL (mmol/L) | 1.66 ± 0.46 | 1.77 ± 0.24 | None |
| TG (mmol/L) | 0.20 ± 0.06 | 0.22 ± 0.06 | None |
| Ga^2+^ (mmol/L) | 2.30 ± 0.06 | 2.31 ± 0.06 | None |
| IP (mmol/L) | 2.57 ± 0.13 | 2.62 ± 0.11 | None |
| Na^+^ (mmol/L) | 147.08 ± 1.08 | 146.63 ± 0.94 | None |
| K^+^ (mmol/L) | 4.38 ± 0.20 | 4.22 ± 0.20 | None |
| Cl^-^ (mmol/L) | 107.48 ± 1.04 | 107.45 ± 1.49 | None |

Note: “None” indicates no statistical difference between the two groups since *p* > 0.05.

**Table S9. The results of organ weight relative to body weight in female rats (%) (*n* = 10)**

| Organs | Control（mean ± SD） | Exp（mean ± SD） | Statistical difference |
| --- | --- | --- | --- |
| Brain | 0.77 ± 0.06 | 0.76 ± 0.04 | None |
| Thymus | 0.17 ± 0.02 | 0.16 ± 0.06 | None |
| Heart | 0.36 ± 0.02 | 0.34 ± 0.02 | None |
| Liver | 2.97 ± 0.23 | 2.82 ± 0.18 | None |
| Spleen | 0.20 ± 0.02 | 0.21 ± 0.03 | None |
| Adrenal | 0.02 ± 0.00 | 0.03 ± 0.01 | None |
| Kidney | 0.78 ± 0.05 | 0.76 ± 0.07 | None |
| Ovary | 0.04 ± 0.01 | 0.05 ± 0.01 | None |
| Uterus | 0.24 ± 0.05 | 0.25 ± 0.06 | None |

Note: “None” indicates no statistical difference between the two groups since *p* > 0.05.

**Table S10. The results of organ weight relative to body weight in male rats (%) (*n* = 10)**

| Organs | Control（mean ± SD） | Exp（mean ± SD） | Statistical difference |
| --- | --- | --- | --- |
| Brain | 0.49 ± 0.04 | 0.50 ± 0.03 | None |
| Thymus | 0.13 ± 0.02 | 0.14 ± 0.04 | None |
| Heart | 0.32 ± 0.02 | 0.32 ± 0.03 | None |
| Liver | 2.69 ± 0.09 | 2.93 ± 0.08 | None |
| Spleen | 0.18 ± 0.03 | 0.21 ± 0.03 | None |
| Adrenal | 0.01 ± 0.00 | 0.01 ± 0.00 | None |
| Kidney | 0.74 ± 0.06 | 0.77 ± 0.06 | None |
| Testis | 0.81 ± 0.07 | 0.81 ± 0.08 | None |
| Epididymises | 0.25 ± 0.02 | 0.26 ± 0.02 | None |

Note: “None” indicates no statistical difference between the two groups since *p* > 0.05.


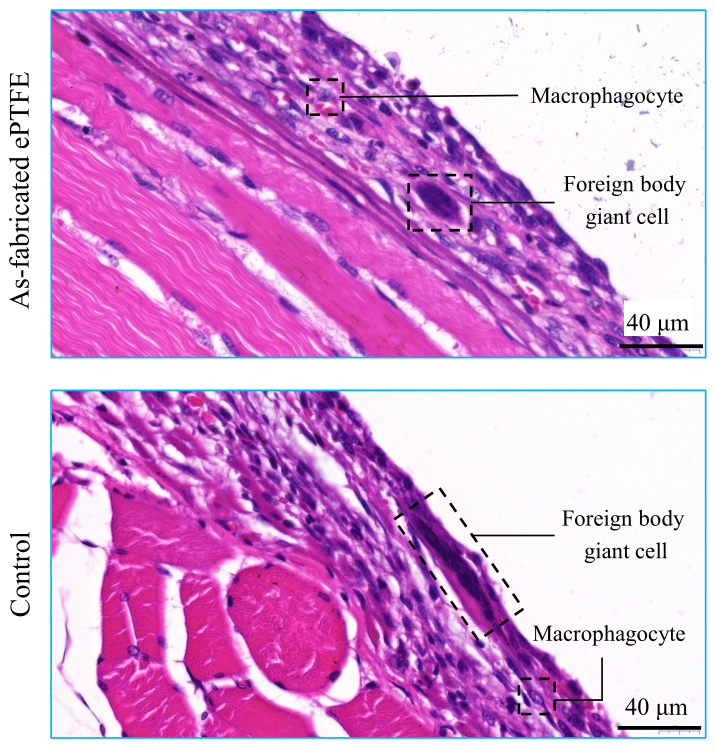


**Figure S6. Optical micrograph of HE-stained slices of the implantation site one week after implantation in rabbits.** Some inflammation cells were scattered in the capsule wall with macrophagocyte and foreign body giant cell observed occasionally.
